# Integrated analysis of transcriptome and metabolome data reveals insights for molecular mechanisms in overwintering Tibetan frogs, *Nanorana parkeri*

**DOI:** 10.3389/fphys.2022.1104476

**Published:** 2023-01-09

**Authors:** Yonggang Niu, Xuejing Zhang, Shengkang Men, Kenneth B. Storey, Qiang Chen

**Affiliations:** ^1^ Department of Life Sciences, Dezhou University, Dezhou, China; ^2^ School of Life Sciences, Lanzhou University, Lanzhou, China; ^3^ Department of Biology, Carleton University, Ottawa, ON, Canada

**Keywords:** transcriptomic, metabolomic, hibernating, metabolism, Nanorana parkeri

## Abstract

*Nanorana parkeri* (Anura, Dicroglossidae) is a unique frog living at high altitude on the Tibetan plateau where they must endure a long winter dormancy at low temperatures without feeding. Here, we presented a comprehensive transcriptomic and metabolomic analysis of liver tissue from summer-active *versus* overwintering *N. parkeri*, providing the first broad analysis of altered energy metabolism and gene expression in this frog species. We discovered that significantly up-regulated genes (2,397) in overwintering frogs mainly participated in signal transduction and immune responses, phagosome, endocytosis, lysosome, and autophagy, whereas 2,169 down-regulated genes were mainly involved in metabolic processes, such as oxidation-reduction process, amino acid metabolic process, fatty acid metabolic process, and TCA cycle. Moreover, 35 metabolites were shown to be differentially expressed, including 22 down-regulated and 13 up-regulated in winter. These included particularly notable reductions in the concentrations of most amino acids. These differentially expressed metabolites were mainly involved in amino acid biosynthesis and metabolism. To sum up, these findings suggest that gene expression and metabolic processes show adaptive regulation in overwintering *N. parkeri*, that contributes to maintaining homeostasis and enhancing protection in the hypometabolic state. This study has greatly expanded our understanding of the winter survival mechanisms in amphibians.

## Introduction

Winter hibernation is an extreme physiological state and the success of hibernation seriously affects the survival, reproduction, and population density of amphibians (Bradford, 1983; Santana et al., 2015). Overwintering amphibians have developed behavioral, physiological, biochemical, and molecular mechanisms to counter numerous environmental stressors that occur during winter (Storey and Storey, 2017). For example, the water frogs *Rana esculenta* and *R. lessonae* often change hibernation sites more than once during winter (Holenweg and Reyer, 2000). Other behaviors, such as overwintering underwater (Tattersall and Ultsch, 2008), digging deep into the soil (Denton and Beebee, 1993), or using existing tunnels to burrow below the frost line (Browne and Paszkowski, 2010), can be effective in circumventing or buffering extreme weather conditions during winter. If these favorable conditions are denied, as in the case in some terrestrially-hibernating amphibians, they have to rely on physiological strategies to cope with the onslaught of subzero temperatures (Storey and Storey, 1986). Two of the most typical ways are freeze tolerance and freeze avoidance that have been widely noted in terrestrially-hibernating ectotherms (Costanzo and Lee, 2013). Multiple biochemical and molecular adaptive strategies have also been documented to enable successful overwintering of hibernators including 1) changes in enzyme activities, gene transcription, and translation (Storey and Storey, 2004), 2) reversible post-transcriptional modifications (e.g., phosphorylation, acetylation) (Dawson et al., 2015), 3) alteration of mitochondrial density and/or characteristics (Guderley and St-Pierre, 2002), 4) generation of protective molecules (e.g., antifreeze proteins, cryoprotectants, and chaperone proteins) (Storey and Storey, 2017), and 5) modifications of membrane lipids (Hazel, 1995). It is well known that animals can actively or passively enter a hypometabolic or dormant state when faced with environmental stresses (e.g., extreme cold and low food availability) that pose serious challenges to their survival (Storey and Storey, 2004).

High-throughput omics approaches have been widely used to explore animal adaptations to abiotic stresses (Ren et al., 2020; Chang et al., 2021; Pei et al., 2021; Zhang et al., 2021; Mo et al., 2022). For instance, transcriptomic analyses were used to assess seasonal adaptive mechanisms in the Chinese alligator (*Alligator sinensis*), and the expression levels of *ADIPO*, *CIRBP* and *TMM27* were up-regulated in the dormant Chinese alligator (Sun et al., 2018). Transcriptomic analyses showed that significantly up-regulated genes were mainly involved in stress responses, whereas down-regulated genes were mainly involved in metabolic depression and shifts in energy utilization in hibernating female Asiatic toads (*Bufo gargarizans*) (Jin et al., 2018). Although transcriptome sequencing can offer gene expression information related to different phenotypes and contribute to functional genomics studies (Salem et al., 2010), phenotypes are also regulated at physiological and biochemical levels, one of which is the metabolic level. These studies are valuable for increasing our understanding of hibernation physiology, but they focused on hibernating rodents, such as *Ictidomys tridecemlineatus*, the thirteen-lined ground squirrel (Nelson et al., 2010; Nelson et al., 2009; D’Alessandro et al., 2017), and Syrian hamsters (*Mesocricetus auratus*) (Gonzalez-Riano et al., 2019). However, hibernation is a highly integrative process and thus there are unique advantages to using a systems biology approach, in conjunction with multi-omics techniques, to explore the underlying mechanisms. For instance, Pei et al. (2021) utilized transcriptomics and metabolomics to characterize the key metabolic pathways associated with cold tolerance in overwintering *Streltzoviella insularis* (Staudinger) (Lepidoptera: Cossidae) larvae. Multi-omics analyses also revealed the unique energy-saving strategy of hibernating Chinese alligators (*Alligator sinensis*) (Lin et al., 2020). Therefore, an integrated application of metabolomics and transcriptomics can provide novel insights for winter hibernation and metabolic depression in overwintering frogs, especially for living at high altitudes where frogs undergo longer periods of dormancy accompanied by multiple stresses, such as extreme cold temperatures, lower oxygen partial pressure, and prolonged starvation. The potential reason was that the winter becomes longer and ambient temperatures and oxygen partial pressures continue to drop as the altitude increases.

*Nanorana parkeri* (Anura, Dicroglossidae), a frog species endemic to the Tibetan Plateau, is widely distributed from 2,850 to 5,100 m above sea level (a.s.l) (Sun et al., 2015). This frog is well-adapted to living at high altitudes and surviving the relatively harsh winters of the region (Ma et al., 2009). The physiological ecology of winter hibernation has been well studied in the Xizang plateau frog, *N. parkeri* (Niu et al., 2022). We found that this species can tolerate a brief freezing within body, and freezing exposure induced an anticipatory up-regulation of antioxidant enzymes in the liver and brain (Niu et al., 2021a; Niu et al., 2021b). A higher level of oxidative stress and lower antioxidant defenses was observed in overwintering *N. parkeri* (Niu et al., 2018). We also examined differences in plasma metabolic profiles in summer and winter (Niu et al., 2021c), but no studies have thoroughly assessed changes in metabolites in liver or revealed the underlying molecular regulatory mechanisms involved. Metabolic depression is crucial for the survival of many species that use dormancy as a survival mechanism, lowering their metabolic rate to minimal levels to endure prolonged exposures to sub-optimal environmental conditions (Storey and Storey, 1990; Guppy and Withers, 1999). However, the molecular mechanisms underlying metabolic depression in overwintering *N. parkeri* are still poorly understood. Investigating the metabolic adaptations of winter hibernation in *N. parkeri* can contribute to revealing the cold hardiness of overwintering ectothermic vertebrates living at high altitude and elucidating the adaptation mechanism of amphibian to the extreme environment (multiple stresses) on the Tibetan plateau.

To further understand biochemical and molecular adjustments in hibernating *N. parkeri*, in the present study, we compared the transcriptomes and metabolomes in the liver of summer-active and overwintering frogs since liver as a significant metabolic organ plays an important role in environmental adaptation. Genes and metabolites associated with the hibernation phenotype were identified and integrated into the metabolic pathways together. This study will provide new insights into the underlying molecular mechanisms of metabolic depression and highlight the significance of an integrative approach.

## Materials and methods

### Animals and sample collection

Adult male *N. parkeri* (*n* = 13 in each season) were collected in mid-July while active and in December, during the hibernating season in Damxung County (30.28° N, 91.05° E; 4,280 m), Tibet, China. Summer-active frogs were collected from breeding ponds and hibernating frogs were collected from small caves underwater in the wild. All frogs collected were healthy. Moreover, both microhabitat temperature in winter and body temperature of hibernating *N. parkeri* were all significantly lower than those in summer as well as a lower dissolved oxygen content in the water in winter (Niu et al., 2022). Frogs with similar body weight (4.0–5.0 g) were used, ensuring uniformity of among samples in both seasons (detailed morphological parameters are presented in Supplementary Table S1). Euthanasia was by decapitation near the capture site. Samples of liver (*n* = 13 for each season) were collected immediately and frozen in liquid nitrogen. Liver tissues (*n* = 3 for each season) were used for transcriptomic analysis and prepared for RNA extraction immediately to reduce the chance of RNA degradation after collection. Remaining frozen liver tissue samples (*n* = 10 for each season) were stored at −80°C until the sampling process was completed for the two seasons and were then used for GC-MS analysis.

### Transcriptome analysis and qRT-PCR validation

Trizol reagent was used to extract total RNA from liver samples (*n* = 3 for each season) followed by testing purity using a NanoDrop 2000 micro spectrophotometer (Thermo Scientific, United States). RNA concentration and integrity were evaluated using an RNA 6000 Nano Kit with an Agilent Bioanalyzer 2,100 (Agilent Technologies, CA, United States). Subsequently, mRNA was enriched with oligo (dT) magnetic beads, followed by addition of fragmentation buffer to cut the mRNA into short fragments. Next, first strand cDNA was produced using random hexamer primers and the mRNA fragments as templates. Subsequent synthesis of second strand cDNA was carried out after adding dNTPs, DNA polymerase I, RNaseH and buffer. A QiaQuick PCR kit was then used to purify double-stranded cDNA, followed by end repair, base A addition and sequencing junction treatment. This was followed by agarose gel electrophoresis to recover target fragments for PCR amplification. A HiSeq 2,500 (Illumina) platform (Annoroad Gene Technology Corporation; Beijing, China) was then used for paired-end 150-bp sequencing. Raw sequence data was deposited in the Genome Sequence Archive (GSA) database (https://ngdc.cncb.ac.cn/gsa/); accession number of PRJCA010139. Data pre-processing and reference-based RNA-Seq analysis was conducted using the BMKCloud service (http://www.biocloud.net/). Raw RNA sequencing data was processed with FastQC (http://www.bioinformatics.babraham.ac.uk/projects/fastqc/) to remove adapter sequences, ambiguous sequences (“N” larger than 10%), or sequences with >50% of low-quality bases. Clean reads were then mapped to the *N. parkeri* reference genome (https://ftp.ncbi.nlm.nih.gov/genomes/all/GCF/000/935/625/GCF_000935625.1_ASM93562v1). Read-count data were analyzed using the DEseq2 package and |Log_2_FoldChange| >1 and a False Discovery Rate (FDR) < 0.01 were used to define differentially expressed genes (DEGs). DEGs were then used for functional enrichment analysis including gene ontology (GO) analysis and Kyoto Encyclopedia of Genes and Genomes (KEGG) assessment. Enrichment analysis of DEGs was conducted with *p* < 0.05 as the threshold. To validate DEG expression levels, we randomly selected and analyzed 13 genes using real time qRT-PCR. Reverse transcription (RT) was then used with HiScript III RT SuperMix for qPCR (Vazyme, China), and qRT-PCR was performed with TransStart Tip Green qPCR SuperMix (TransGen Biotech, China) using a Bio-Rad CFX (Bio-Rad, United States). Primer Premier 5.0 was used to design primer sequences that were then synthesized by Sangon Biotech Co., Ltd. (Shanghai, China). Details of primers are shown in Supplementary Table S2. Relative expression levels were determined with the 2^−ΔΔCt^ method using *GAPDH* as an internal control gene (Livak and Schmittgen, 2001). All samples were run in triplicate and each group contained three biological replicates.

### Metabolomics analysis

Samples of liver tissue (50 mg each, *n* = 10 for each group) were added to Eppendorf tubes with 1 ml chloroform/methanol/water (v:v:v = 2:5:2) solvent, containing 5 μg mL^−1^ l-norleucine). Tissue samples were homogenized (while being held in an ice bath) using a TissueLyser (JX-24, Jingxin, Shanghai) with two zirconia beads for 3 min at 30 Hz. Next, an aliquot of internal standard (10 μL containing 0.05 mg ml^−1^ ^13^C-^15^N-l-isoleucine) was added into 100 μL of supernatant, followed by evaporation to dryness with a nitrogen stream. The dry residue was re-dissolved in 30 μL of 20 mg ml^−1^ methoxyamine hydrochloride with pyridine, followed by incubation for 90 min at 37 °C. Then a 30 μL volume of N, O-bis (trimethylsilyl) trifluoroacetamide (BSTFA; containing 1% TMCS) was added followed by derivatization for 60 min at 70 °C and then GC-MS metabolomics analysis. Quality control (QC) samples were prepared by mixing equal aliquots of twenty samples from both seasons and then analyzed as above. All GC-MS metabolomics analysis used an Agilent 7890A gas chromatography system together with an Agilent 5975C inert MSD system (Agilent Technologies Inc, CA, United States). A Rxi-5 Sil MS fused-silica capillary column (30 m × 0.25 mm × 0.25 μm) (Agilent J&W Scientific, Folsom, CA, United States) was used to separate derivatives. Helium (>99.999%) was the carrier gas with a flow rate of 1 ml min^−1^ going through the column. Injection volume was 1 μl, solvent delay time was 6 min, and the initial oven temperature was 70 °C for 2 min, then ramped to 160°C (with a rate of 6°C min^−1^). Next, temperature was raised to 240°C at a rate of 10 °C min^−1^, followed by a rise to 300°C (20 °C min^−1^) with a final hold at 300°C for 6 min. Temperatures of the injector, transfer line, and electron impact ion source were 250°C, 250°C, and 230°C, respectively. The impact energy was 70 eV, and data was collected in a full scan mode (m/z 50–600). Peak picking, alignment, deconvolution and additional processing of raw GC-MS data was as in previous protocols (Gao et al., 2010). Deconvolution of mass spectra from raw GC-MS data was carried out with AMDIS software, and purified mass spectra data were matched automatically with the in-house standard library (Profleader Biotech Co., Ltd, Shanghai, China) including mass spectra and retention time using the Golm Metabolome Database, and Agilent Fiehn GC/MS Metabolomics RTL Library.

### Statistical analysis of metabolomics data

Final data were imported into SIMCA (V14.1, Sartorius Stedim Data Analytics AB, Umea, Sweden) software for multivariate statistical analysis that included PCA (principal component analysis) and OPLS-DA (orthogonal projections to latent structures discriminant analysis) followed by statistical analysis using Student’s *t*-test. Significantly changed metabolites (SCMs) were identified based on values for variable importance for the projection (VIP >1) with *p* < 0.05. SCMs were then used for relevant biological pathway analysis with MetaboAnalyst 5.0 (https://www.metaboanalyst.ca/). Pathways with impact scores >0.05 and -ln (*p*-value) > 1.0 were identified as potentially affected.

### Integrated analysis of metabolome and transcriptome

The DEGs and SCMs were simultaneously mapped to major metabolic pathways, including glycolysis, gluconeogenesis, TCA cycle, oxidative phosphorylation, fatty acid and amino acid metabolism, as well as the pentose phosphate pathways, to demonstrate the changes occurring in metabolic pathways from a global perspective.

## Results

### Transcriptome analysis

In total of 144, 367, 762 and 170, 882, 348 clean reads were generated from the summer and winter group, respectively (Supplementary Table S3). Quality analysis showed that summer and winter groups had a mean of 45.31% and 46.62% GC content of clean reads, and a Q30 of 91.84% and 89.81%, respectively. The average map rate to the genome of *N. parkeri* was 87.93% for the summer group and 83.86% for the winter group, respectively (Supplementary Table S3). Pearson correlation analysis showed a high correlation between the biological replicates (Supplementary Figure S1), reflecting the reliability of the test. A total of 4,566 differentially expressed genes (DEGs) were found in liver between summer and winter, in which 2,397 genes showed a significant up-regulation and 2,169 genes were down-regulated in winter compared with summer (Supplementary Figure S2, Supplementary Table S4).

**FIGURE 1:**
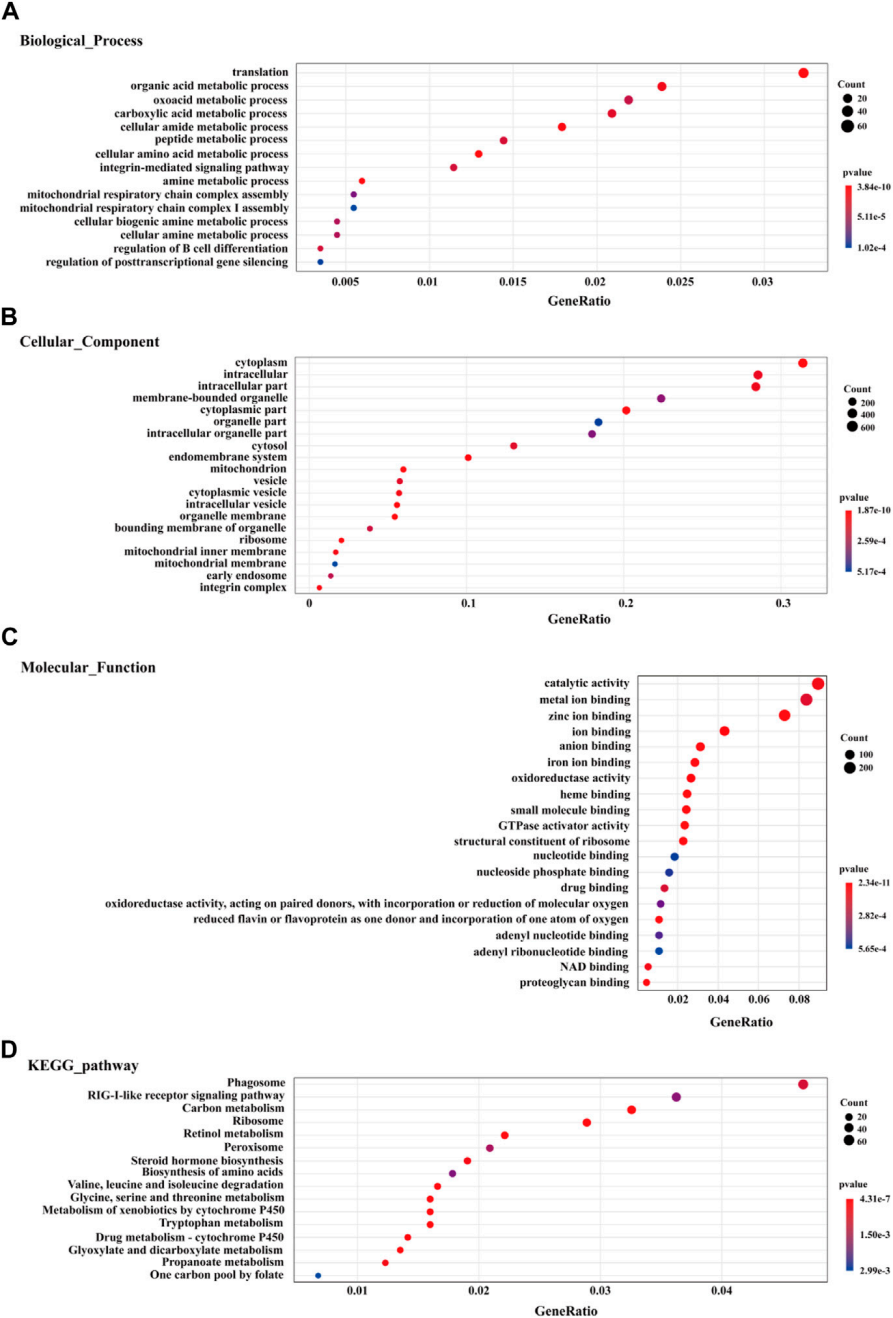
The top 20 enriched GO terms (biological process **(A)**, cellular component **(B)**, and molecular function **(C)** and KEGG pathway enrichment analysis **(D)** for DEGs in liver of winter-collected *N. parkeri* compared with summer-collected individuals (*p* < 0.05). The color shades and circle size represent different *p* values and gene count, respectively.

Using GO enrichment analysis, the data showed that the top GO term among all DEGs of the biological process category were “translation”, “metabolic process”, “biological regulation”, and “multicellular organismal process” (Figure 1A). In the cellular component category, the GO terms “cytoplasm”, “intracellular”, “organelle part”, “mitochondrion”, “vesicle” and “ribosome” were significantly enriched (Figure 1B). the GO terms “catalytic activity”, “binding”, and “oxidoreductase activity” were the most represented among the molecular function category (Figure 1C). The top 20 GO terms for enriched up-regulated genes are exhibited in Supplementary Figure S3. In the biological process category, the most up-regulated genes were linked to signal transduction (GO: 0007165), regulation of response to stimulus (GO: 0048583), immune response (GO: 0006955), and immune system process (GO: 0002376). In the molecular function category, most up-regulated genes were enriched in ion binding (GO: 0043167), protein binding (GO: 0005515), and GTPase activator activity (GO: 0005096). In the cellular component category, the most up-regulated genes were enriched in cytoplasm (GO: 0005737), intracellular (GO: 0005622), cell periphery (GO: 0071944), and vesicle (GO: 0031982) (Supplementary Figure S3). Among down-regulated genes, the top 20 enriched GO terms are exhibited in Supplementary Figure S4. For biological process, down-regulated genes primarily participated in organic acid metabolic process (GO: 0006082), oxidation-reduction (GO: 0055114), tricarboxylic acid cycle (GO: 0006099), cell amino acid metabolic process (GO: 0006520), and fatty acid metabolic process (GO: 0006631). Under molecular function, down-regulated genes were mainly enriched in catalytic activity (GO: 0003824), RNA binding (GO: 0003723), and oxidoreductase activity (GO: 0016491). For the cellular component category, the down-regulated genes were mainly enriched in cytoplasm (GO: 0005737), mitochondrion (GO: 0005739), and intracellular organelle lumen (GO: 0070013) (Supplementary Figure S4).

KEGG pathway enrichment analysis found enriched DEGs in the pathways of phagosome (ko04145), carbon metabolism (ko01200), ribosome (ko03010), peroxisome (ko04146), and biosynthesis of amino acids (ko01230) (Figure 1D). Up-regulated genes were also significantly enriched in the immune defense-related pathways, such as phagosome (ko04145), RIG-I-like receptor signaling pathway (ko04622), endocytosis (ko04144), lysosome (ko04142), autophagy-animal (ko04140), and intestinal immune network for IgA production (ko04672) (Supplementary Figure S5A). The expression levels of DEGs in immune response and damage repair mechanisms (e.g. antioxidant defense, phagosome, endocytosis, lysosome, and autophagy) are visualized in heat map diagrams (Supplementary Figures S6,S7). However, the down-regulated genes were significantly enriched mainly in metabolism-related pathways (Supplementary Figure S5B), such as carbon metabolism (ko01200), valine, leucine and isoleucine degradation (ko00280), tryptophan metabolism (ko00380), fatty acid degradation (ko00071), biosynthesis of amino acids (ko01230), primary bile acid biosynthesis (ko00120), fatty acid metabolism (ko01212), citrate cycle (TCA cycle) (ko00020), oxidative phosphorylation (ko00190), and glycolysis/gluconeogenesis (ko00010). The expression levels of DEGs involved in metabolic processes are visualized in heat map diagrams (Supplementary Figure S8).

**FIGURE 2:**
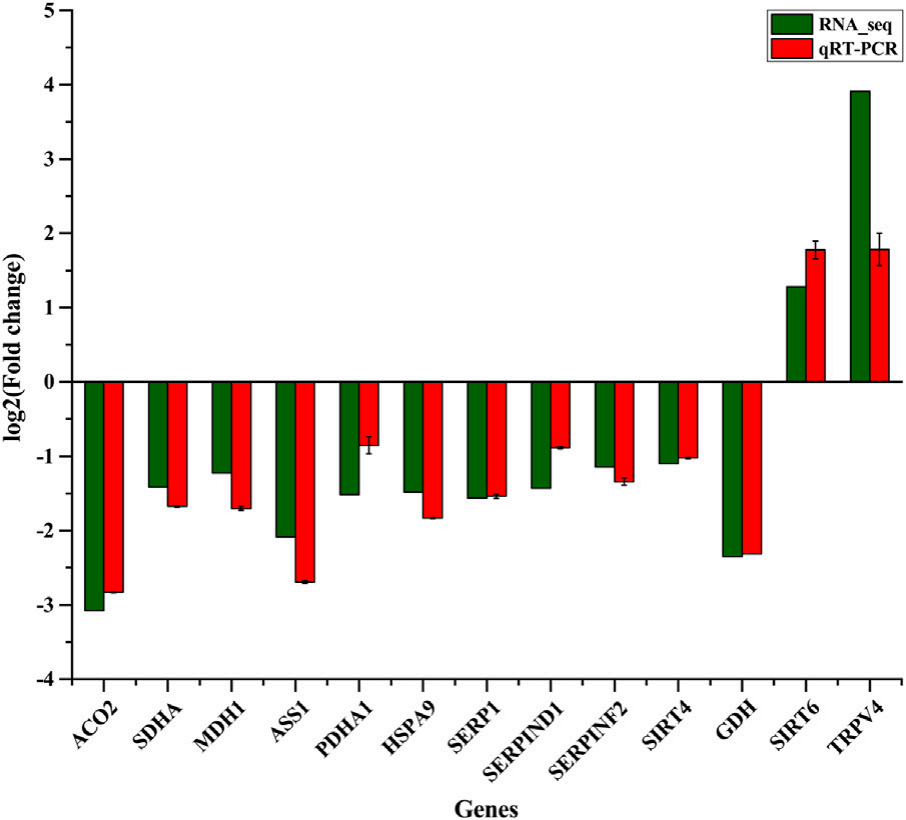
Thirteen DEGs were validated by comparison of RNA-Seq and qRT-PCR in liver of winter-collected *N. parkeri* compared with summer-collected individuals.

To confirm the results of RNA-seq, RT-qPCR was applied to assess the expression of 13 randomly selected genes including *ACO2*, *SDHA*, *MDH1*, *ASS1*, *GDH*, *PDHA1*, *HSPA9*, *SERP1*, *SERPIND1*, *SERPINF2*, *SIRT4*, *SIRT6*, and *TRPV4*. These genes are involved in the TCA cycle, ornithine cycle (urea cycle), amino acid metabolism, and chaperones. Expression levels of *ACO2*, *SDHA*, *MDH1*, *ASS1*, *GDH*, *PDHA1*, *HSPA9*, *SERP1*, *SERPIND1*, *SERPINF2*, and *SIRT4* genes decreased significantly whereas *SIRT6* and *TRPV4* genes were significantly up-regulated in the winter as compared to summer (Figure 2). Overall, the analysis *via* RT-qPCR showed that up- and down-regulated genes agreed well with results from RNA-sequencing, showing high reliability of RNA-sequencing data.

### Metabolome analysis

**FIGURE 3:**
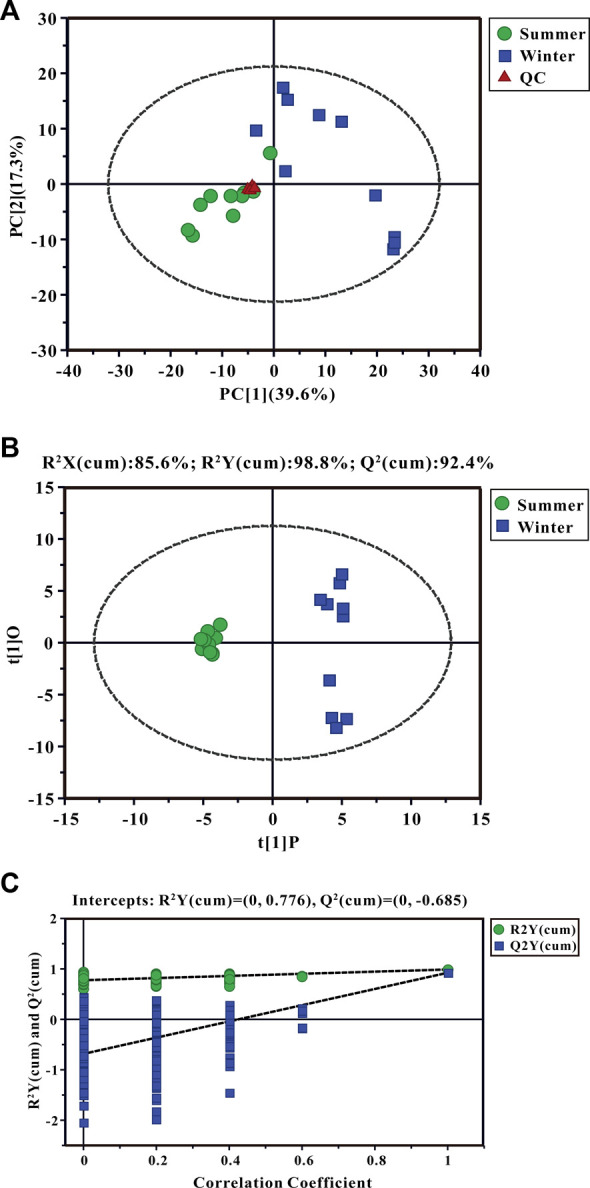
Principal component analysis (PCA) score plots **(A)**, the orthogonal projection to latent structures discriminant analysis (OPLS-DA) scores plots **(B)**, and permutation tests **(C)** for liver metabolites. Three groups are distinguished: summer-collected frogs, green; winter-collected frogs, blue; and Quality Control (QC) samples, red.

**FIGURE 4:**
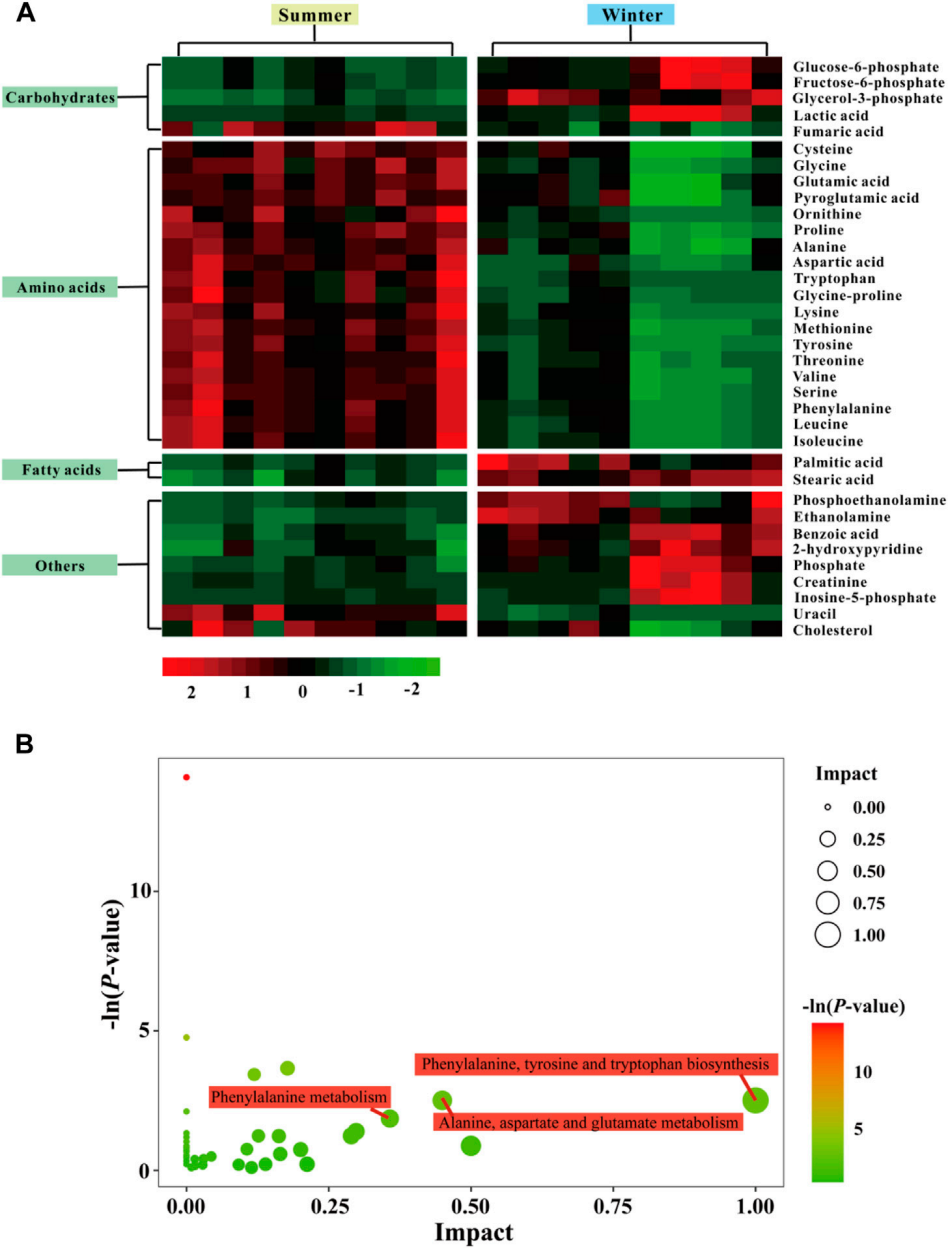
Heat map of identified differentially expressed metabolites in the liver from summer- and winter-collected *N. parkeri* **(A)**. The impact score (0,1) indicates the pathway topological importance of the metabolites. The color and size of each circle is based on *p* values and pathway impact values, respectively. Pathway analysis of differential metabolites in liver **(B)**; red color indicates highly expressed metabolites, and green indicates low expressed metabolites.

Using GC-TOF/MS, 377 valid peaks were extracted and a total of 151 metabolites were identified from public databases and an in-house standard library. A plot of PCA scores found that five QC samples were densely distributed with considerable separations between summer and winter groups. This suggested good stability of the PCA model and changes in metabolic physiology during winter (Figure 3A). To maximize the discrimination of metabolic patterns in liver between summer and winter (Figure 3B), the OPLS-DA model was used and the score plot showed a clear separation of samples between winter and summer groups. The estimated goodness of fit of R^2^Y and the goodness of prediction of Q^2^Y values were 98.8% and 92.4%, respectively, and all were stable and effective for fitness and prediction. Permutation testing showed that the R^2^ *Y*-intercept and Q^2^-intercept were 0.78 and -0.69, respectively (Figure 3C). These parameters indicate robustness and that no over-fitting occurred using the OPLS-DA model. A total of 35 significantly different metabolites were screened: 13 were up-regulated and 22 were down-regulated during winter as compared to summer. Heat map clustering analysis showed the relative amounts of metabolites that were significantly differentially expressed and their relationships in the two groups (Figure 4A). Differentially expressed metabolites are listed in Supplementary Table S5, mainly including carbohydrates, lipids, amino acids, and their derivatives. Based on both -ln (*p*-value) > 1.0 and pathway impact scores >0.05, the significant metabolic pathways affected by hibernation were screened, mainly including phenylalanine, tyrosine and tryptophan biosynthesis, alanine, aspartate and glutamate metabolism, phenylalanine metabolism, glycine, serine and threonine metabolism, arginine and proline metabolism, arginine biosynthesis, starch and sucrose metabolism, glycerophospholipid metabolism, and glutathione metabolism (Figure 4B).

### Integrative analysis of transcriptome and metabolome

**FIGURE 5:**
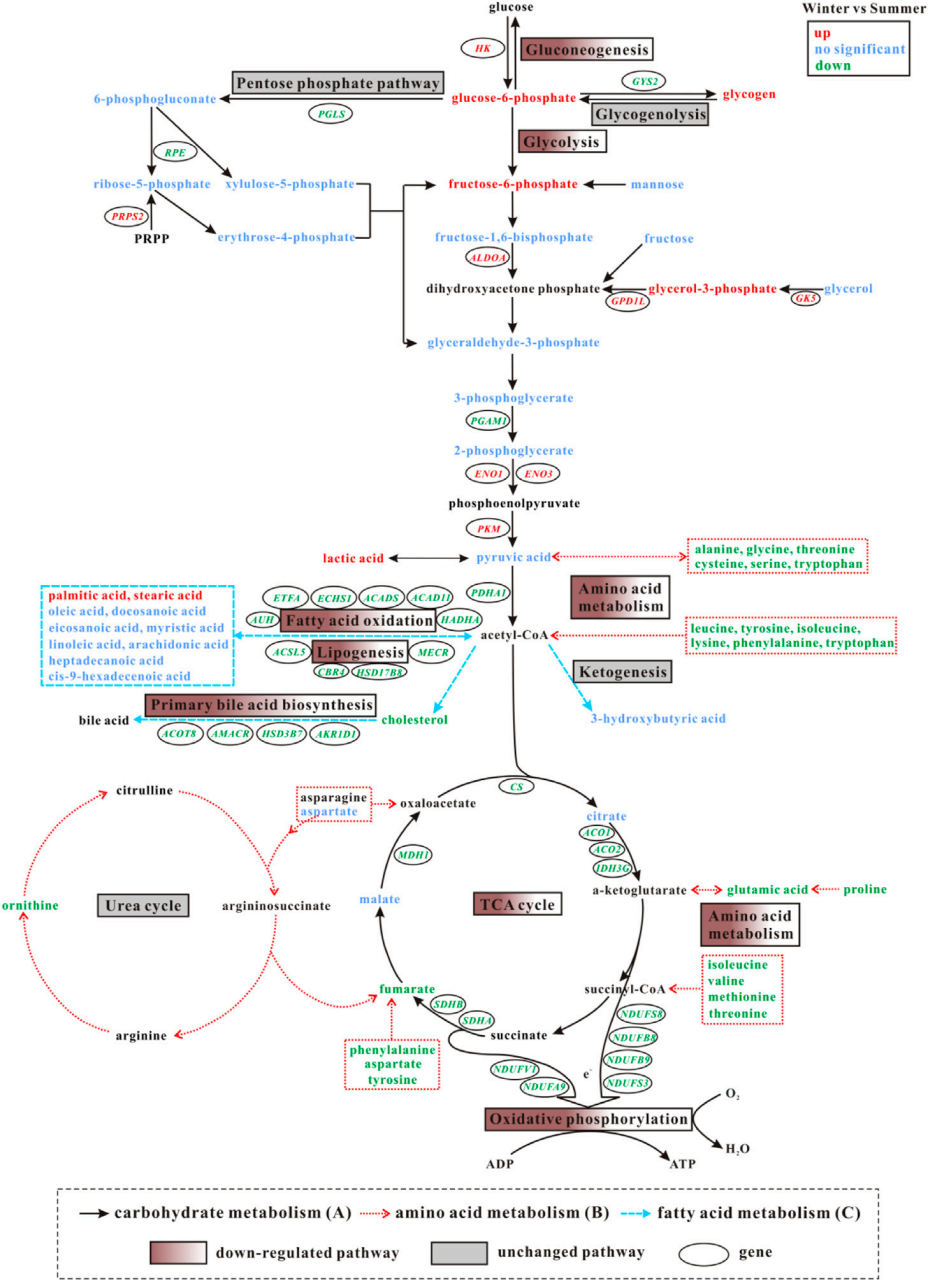
Primary changes in gene expression and metabolites in pathways participating in energy metabolism in liver of *N. parkeri* between the two seasons (winter vs. summer): carbohydrate metabolism **(A)**, amino acid metabolism **(B)**, fatty acid metabolism **(C)**. Red color indicates up-regulated genes and metabolites, green indicates down-regulated genes and metabolites, and blue indicates no significant change in metabolite level.

Transcriptome and metabolome analysis showed changes in primary metabolic pathways (Figure 5). Glycolysis/gluconeogenesis, fatty acid oxidation, lipogenesis, primary bile acid biosynthesis, TCA cycle, amino acid metabolism, and oxidative phosphorylation all were down-regulated in liver from winter *versus* summer frogs, whereas no significant changes were found in pentose phosphate pathway, glycogenolysis, ketogenesis, and urea cycle.

## Discussion

This study is the first investigation to identify key genes, metabolites, and metabolic pathways associated with winter hibernation in *N. parkeri* using integrated transcriptomic and metabolomic analyses. We found that *N. parkeri* adapts to winter conditions mainly by reducing most energy metabolic processes and enhancing immune responses and damage repair.

### Carbohydrate metabolism

It is well known that amphibians usually accumulate large substrate reserves (such as liver glycogen) during pre-hibernation to provide energy for overwintering and to fuel reproductive activities after arousal in the early spring (Singh and Sinha, 1989). Higher hepatic glycogen reserves were observed in overwintering *N. parkeri*, supporting prolonged periods of hibernation (Niu et al., 2022). We found that the expression of genes (*GYS2* and *LOC108789874*) encoding glycogen synthase and UDP-glucose pyrophosphorylase 2 (*UGP2*), respectively, were significantly down-regulated, whereas the expression of genes encoding glycogen phosphorylase (*GYPL*), glycogen debranching enzyme (*AGL*), phosphoglucomutase (*PGM1*, *PGM2*, *PGM3*), and glucose-6-phosphatase (*G6PC1*, *G6PC3*) were not significantly changed in the liver. These results suggest that hepatic glycogen synthesis is inhibited but glycogen breakdown is not affected during hibernation. Moreover, metabolomic data suggest that glucose-6-phosphate (G6P) and fructose-6-phosphate were significantly accumulated and this may be derived from the breakdown of hepatic glycogen. Similarly, both G6P and fructose 2,6-bisphosphate increased significantly in liver of freezing-exposed turtles, *Chrysemys picta marginata* (Storey et al., 1988). This is consistent with the persistence of glycogen breakdown as an anaerobic fuel and lactate production as the end product during hibernation.

Adenosine triphosphate (ATP) can be produced by the degradation of carbohydrates through glycolysis, TCA cycle, and oxidative phosphorylation. In this study, the transcripts of genes involved in these pathways were significantly down-regulated in winter, consistent with the metabolic depression in hibernating *N. parkeri* (Niu et al., 2020). A similar finding was reported in the hibernating Chinese alligator, genes encoding 6-phosphofructokinase (6-PFK, the rate-limiting enzyme of glycolysis), citrate synthase (a rate-limiting enzyme of the TCA cycle), isocitrate dehydrogenase (*IDH*), and oxoglutarate dehydrogenase (*OGDH*) were significantly down-regulated during hibernation (Lin et al., 2020). These changes imply the need to readjust intracellular ATP production and hydrolysis in the hibernating state. However, mRNA levels of hexokinase (*LOC108795789*, *LOC108788971*), pyruvate kinase (*PKM*), enolase (*EN O 1*, *EN O 3*), l-lactate dehydrogenase B (*LOC108788318*) (*p* < 0.05), and fructose-bisphosphate aldolase (*ALDOA*) showed a significant increase in hibernating *N. parkeri*. Increased gene expression of *LOC108788318* coincided with elevated lactate dehydrogenase activity in winter (Niu et al., 2020). Moreover, metabolomics analysis showed a significant accumulation of lactate content in liver. These results suggest that anaerobic glycolysis remains active during hibernation, and that it can at least provide ATP for energy-consuming processes such as protein hydrolysis, albeit at a much lower efficiency than aerobic metabolic pathways. Similarly, European common lizards (*Lacerta vivipara*) showed a significant augmentation of anaerobic metabolism and lactate accumulation in winter, which stems from an activation of the lactate fermentation pathway (Voituron et al., 2000). The results of the present study suggest that *N. parkeri* develops a good tolerance for lactate accumulation and that metabolic depression primarily suppresses aerobic metabolism rather than anaerobic pathways.

### Lipid and fatty acid metabolism

Lipids are thought to be the other major fuel reserve for metabolism during hibernation (Tattersall and Ultsch, 2008). Previous studies have demonstrated that amphibians store large amounts of lipids in early fall and that these energy reserves are depleted during the winter and spring spawning periods (Lu et al., 2008). Triglycerides are cleaved by lipase into fatty acids and glycerol that can be further metabolized intracellularly or released into the circulation. In this study, the expression level of *LIPC*, a gene encoding triglyceride lipase, was significantly down-regulated in winter. Moreover, the expression of genes encoding proteins of the fatty acid β-oxidation pathway were also significantly decreased, such as *HADHA* (ecoding β-hydroxyacyl coenzyme A dehydrogenase), *AUH*, *ETFA*, and *ACAD11*. These results indicate that both lipid degradation and fatty acid oxidation processes were significantly reduced in the liver of hibernating *N. parkeri*. Two saturated fatty acids (palmitic acid and stearic acid) showed a significant increase in winter, whereas oleic acid, linoleic acid, and docosanoic acid did not change. Similar results were found in *R. esculenta*, where saturated fatty acid content in the liver increased significantly in winter (Scapin et al., 1990). Palmitic acid and stearic acid are the main precursors for *de novo* synthesis of fatty acids, thus higher levels of palmitic and stearic acid in winter are mainly due to the suppression of fatty acid biosynthetic pathways. The transcriptome results were also consistent with this since genes involved in fatty acid biosynthesis (e.g. *HSD17B8*, *ACSL5*, *CBR4*, and *MECR*) were downregulated during hibernation. Phosphoethanolamine (a derivative of ethanolamine) and glycerol-3-phosphate both participate in the biosynthesis of glycerophospholipids, which is a major component of biological membranes. These three metabolites showed a significant increase in winter. In addition, it has been found that phosphoethanolamine can inhibit the function of mitochondria (Modica-Napolitano and Renshaw, 2004), and thus accumulation of phosphoethanolamine may also facilitate metabolic inhibition in overwintering *N. parkeri*. Similar results were found in a study of the fish (*Perccottus glehni*) where phosphoethanolamine levels in the brain were 94 times higher in winter than in summer. This large change indicates low temperature adaptive modification of membrane phospholipids (Karanova, 2016). Glycerol 3-phosphate can be derived from dihydroxyacetone phosphate or glycerol, catalyzed by glycerol 3-phosphate dehydrogenase or glycerol kinase, respectively. The mRNA levels of glycerol-3-phosphate dehydrogenase (*GPD1L*) and glycerol kinase (*GK5*) were significantly up-regulated in hibernating *N. parkeri* and this can suggest that enhanced glycerol 3-phosphate levels were mainly due to its increased synthesis. Moreover, our previous study showed an increased level of glycerol-3-phosphate and glycerol in the liver of *N. parkeri* after freezing exposure (Niu et al., 2021a). Indeed, glycerol is used as a cryoprotectant by many freeze-tolerant and freeze-avoiding ectotherms including the marine fish, *Osmerus mordax* (Driedzic et al., 2006) and grey tree frogs, *Hyla versicolor* (Storey and Storey, 1985) and *H. chrysoscelis* (Zimmerman et al., 2007). Therefore, the large accumulation of glycerol-3-phosphate in winter may be a preparatory mechanism that can allow a rapid synthesis of glycerol when sudden freezing episodes occur. Overall, these results suggest that fatty acid metabolism was significantly attenuated during the winter.

### Amino acid metabolism

Our previous results have shown that levels of most amino acids are significantly lower in plasma of hibernating *N. parkeri* (Niu et al., 2021c), and the present results from liver metabolomics validates those changes. Similarly, levels of amino acids in plasma were also lower in hibernating European common lizards (*Lacerta vivipara*) (Voituron, 2000). The significant decrease in essential amino acid content during hibernation was related to the lack of food, and the lower levels of non-essential amino acid was mainly due to inhibition of the amino acid biosynthesis process. The carbon skeletons for amino acid synthesis are mainly derived from TCA cycle and glycolysis. In this study, most genes involved in these pathways were down-regulated significantly. Moreover, the expression of enzymes participating in amino acid synthesis and metabolism, such as glutamate dehydrogenase (*LOC108792950*), glutamic-oxaloacetic transaminase (*GOT1*), glutamine synthetase (*LOC108794381*), and asparagine synthetase (*ASNSD1*), decreased significantly in hibernating *N. parkeri*. Gluconeogenesis and protein synthesis, as two major routes of amino acid consumption, were also depressed during torpor (Gehnrich and Aprille, 1988). Our findings showed that gene expression of the glycolysis/gluconeogenesis pathways in the differential gene sets were suppressed, but most differentially expressed genes involved in protein turnover were up-regulated significantly. Therefore, the decrease in amino acid pool is mainly due to fasting and the inhibition of amino acid synthesis. These results imply that homeostasis of synthesis/degradation of amino acids and their derivatives was intensely influenced by wintertime stresses. In terrestrial hibernating amphibians, carbamoyl phosphate synthase (*CPS1*; a rate-limiting enzyme of urea cycle) is normally upregulated to synthesize large amounts of urea, which acts as a cryoprotectant and osmoregulator in response to freezing and dehydration stress during winter (Storey and Storey, 2017). However, transcriptomic data showed no significant change in the expression level of *CPS1*, combining with our previous results of lower urea levels in hibernating *N. parkeri* (Niu et al., 2022), which emphasize a significant down-regulation of amino acid metabolism.

### Immune responses and damage repair mechanisms

Previous studies have reported that hibernation significantly affects the immune system of ectotherms (Maniero and Carey, 1997), including prolonging the induction time of immune responses and allograft rejection time (Lin and Rowlands, 1973), suppressing bone marrow lymphocyte proliferation (Šíma et al., 1981), and reducing the rate and level of antibody production (Cone and Marchalonis, 1972). For instance, desert tortoises (*Gopherus agassizii*) has a lower plasma bactericidal activity in winter, which was a good indicator of innate immunity (Sandmeier et al., 2016). Immune responses cannot be induced at low temperatures during hibernation, which may be due to the thermal sensitivity of lymphocytes and the decrease in lymphocyte numbers (Wright and Cooper, 1981). Therefore, a large up-regulation of immune response-related genes, such as *TNFRSF9* (also called CD137), *TNFAIP8*, *TNFRSF1A*, and *LTB*, in overwintering *N. parkeri* is a compensatory adjustment to reduced lymphatic numbers and thermal sensitivity caused by low temperatures in winter. Similar results were observed in *Ostrinia furnacalis* (Chen et al., 2019), where some immune related genes (such as *PGRPs*, *SPs*, and *AMPs*) were up-regulated in the 8°C treated group compared to 40°C treated group. Our previous study showed that winter-collected *N. parkeri* has a higher bacteria-killing ability of plasma than summer-collected frogs (Niu et al., 2022). These results demonstrated that the immune system is activated in overwintering *N. parkeri*. The up-regulated DEGs involved in immune function and infectious diseases (e.g., *salmonella* infection and *tuberculosis*) in winter probably reflects the seasonal abundance cycle of microbial pathogens, and prolonged overwintering underwater also increases the risk of invasion by waterborne pathogens. Similarly, the prevalence of *Batrachochytrium dendrobatidis* (*Bd*, an amphibian chytrid fungus) infection in *Litoria aurea* was lowest in summer but highest in winter. Both infection load and prevalence decreased with increasing temperature (Garnham et al., 2022). Moreover, *Bd* loads of infected frogs, *R. pipiens*, were 12 times higher in winter than that in summer (Le Sage et al., 2021). Massive amphibian mortality related to fungal pathogens is thought to occur mainly during the cold season (winter) (Rumschlag and Boone, 2018). Therefore, up-regulated immune responses may provide protection for *N. parkeri* from prolonged wintertime stresses.

To eliminate damaged macromolecules and organelles, organisms can utilize efficient repair and removal mechanisms, such as autophagy and the ubiquitin proteasome system (Davies, 2001; Lee et al., 2012). Autophagy is a degradation pathway that can remove intracellular pathogens, damaged organelles, and protein aggregates, in response to stimulation by cellular or environmental stresses (Mizushima, 2007). Autophagy plays an important role in maintaining cellular and tissue homeostasis under many physiological conditions such as aging, immunity, and metabolic stress (Mizushima, 2007; Levine et al., 2011). In this study, expression of most genes involved in autophagy was significantly upregulated in winter, such as *ATG9A*, *ATG14*, *ATG13*, and *ATG4B*. Similar results were found in Chinese soft-shelled turtles (*P. sinensis*), where the autophagy-related gene (*ATG7*), lysosomal-associated membrane protein 1 (*LAMP1*), and microtubule-associated protein light chain (*LC3*) were up-regulated significantly during hibernation (Vistro et al., 2019). Fibrinogen is involved in many biochemical processes in living organisms. Previous studies have reported that fibrinogen synthesis was up-regulated in the liver of wood frogs after freezing exposure (Storey, 1990; Cai and Storey, 1997). Moreover, fibrinogen expression also showed a significant increase in the bullfrog *R. catesbeiana* (Wu et al., 2002). These results suggest that fibrinogen is critical in hibernation-related freeze tolerance and in the damage repair response. We found that the expression levels of genes encoding fibrinogen, such as *LOC108800668*, *LOC108788246*, *ANGPTL2*, *LOC108800667*, *LOC108800665*, *FGL2*, and *ANGPTL3*, were all significantly up-regulated, which may be a protective effect contributing to cold survival of *N. parkeri* during hibernation. Overall, upregulation of these damage repair-related gene expressions is beneficial for the survival of overwintering frogs under an extreme physiological state.

## Conclusion

In conclusion, this study is the first to investigate the winter hibernation in the Xizang plateau frog, *N. parkeri*, combining transcriptomics with metabolomics analysis. We found that genes involved in energy metabolic processes (e.g., glycolysis, gluconeogenesis, TCA cycle, oxidative phosphorylation, amino acid and fatty acid metabolism) were down-regulated in winter hibernation, while most genes related to immune responses and damage repair mechanisms (e.g., phagosome, endocytosis, lysosome, and autophagy) were up-regulated. Most amino acids in liver were significantly reduced in winter. These coordinated changes in gene expression and metabolites observed in *N. parkeri* are crucial for stabilizing macromolecules and promoting long-term survival in the hypometabolic state. Overall, our findings help to expand the knowledge and understanding of the complex regulatory mechanisms that are used by ectothermic vertebrates to respond to stressful environments during the winter.

## Data Availability

The datasets presented in this study can be found in online repositories. The names of the repository/repositories and accession number(s) can be found in the article/Supplementary Material.

## References

[B1] Bradford D. F. (1983). Winterkill, oxygen relations, and energy metabolism of a submerged dormant amphibian, *Rana muscosa* . Ecology 64, 1171–1183. 10.2307/1937827

[B2] Browne C. L. Paszkowski C. A. (2010). Hibernation sites of Western toads (*Anaxyrus boreas*): Characterization and management implications. Herpetol. Conserv. Bio. 5, 49–63.

[B3] Cai Q. Storey K. B. (1997). Freezing-induced genes in wood frog (*Rana sylvatica*): Fibrinogen upregulation by freezing and dehydration. Am. J. Physiol-Reg. I. 272, R1480–R1492. 10.1152/ajpregu.1997.272.5.R1480 9176340

[B4] Chang J. Pan Y. Liu W. Xie Y. Hao W. Xu P. (2021). Acute temperature adaptation mechanisms in the native reptile species *Eremias argus* . Sci. Total. Environ. 818, 151773. 10.1016/j.scitotenv.2021.151773 34808164

[B5] Chen K. Tang T. Song Q. Wang Z. He K. Liu X. (2019). Transcription analysis of the stress and immune response genes to temperature stress in *Ostrinia furnacalis* . Front. Physiol. 10, 1289. 10.3389/fphys.2019.01289 31681003 PMC6803539

[B6] Cone R. E. Marchalonis J. J. (1972). Cellular and humoral aspects of the influence of environmental temperature on the immune response of poikilothermic vertebrates. J. Immunol. 108, 952–957. 10.4049/jimmunol.108.4.952 5063311

[B7] Costanzo J. P. Lee R. E. (2013). Avoidance and tolerance of freezing in ectothermic vertebrates. J. Exp. Biol. 216, 1961–1967. 10.1242/jeb.070268 23678097

[B8] D’Alessandro A. Nemkov T. Bogren L. K. Martin S. L. Hansen K. C. (2017). Comfortably numb and back: Plasma metabolomics reveals biochemical adaptations in the hibernating 13-lined ground squirrel. J. Proteome. Res. 16, 958–969. 10.1021/acs.jproteome.6b00884 27991798

[B9] Davies K. J. (2001). Degradation of oxidized proteins by the 20S proteasome. Biochimie 83, 301–310. 10.1016/s0300-9084(01)01250-0 11295490

[B10] Dawson N. J. Katzenback B. A. Storey K. B. (2015). Free-radical first responders: The characterization of CuZnSOD and MnSOD regulation during freezing of the freeze-tolerant north American wood frog, *Rana sylvatica* . Biochim. Biophys. Acta Gen. Subj. 1850, 97–106. 10.1016/j.bbagen.2014.10.003 25316288

[B11] Denton J. S. Beebee T. J. (1993). Summer and winter refugia of natterjacks (*Bufo calamita*) and common toads (*Bufo bufo*) in Britain. Herpetol. J. 3, 90–94.

[B12] Driedzic W. R. Clow K. A. Short C. E. Ewart K. V. (2006). Glycerol production in rainbow smelt (*Osmerus mordax*) may be triggered by low temperature alone and is associated with the activation of glycerol-3-phosphate dehydrogenase and glycerol-3-phosphatase. J. Exp. Biol. 209, 1016–1023. 10.1242/jeb.02086 16513927

[B13] Gao X. Pujos-Guillot E. Sébédio J. L. (2010). Development of a quantitative metabolomic approach to study clinical human fecal water metabolome based on trimethylsilylation derivatization and GC/MS analysis. Anal. Chem. 82, 6447–6456. 10.1021/ac1006552 20669995

[B14] Garnham J. I. Bower D. S. Stockwell M. P. Pickett E. J. Pollard C. J. Clulow J. (2022). Seasonal variation in the prevalence of a fungal pathogen and unexpected clearance from infection in a susceptible frog species. Dis. Aquat. Organ 148, 1–11. 10.3354/dao03628 35142293

[B15] Gehnrich S. C. Aprille J. R. (1988). Hepatic gluconeogenesis and mitochondrial function during hibernation. Comp. Biochem. Physiol. B 91, 11–16. 10.1016/0305-0491(88)90107-1 3197388

[B16] Gonzalez-Riano C. León-Espinosa G. Regalado-Reyes M. García A. DeFelipe J. Barbas C. (2019). Metabolomic study of hibernating syrian hamster brains: In search of neuroprotective agents. J. Proteome. Res. 18, 1175–1190. 10.1021/acs.jproteome.8b00816 30623656

[B17] Guderley H. St-Pierre J. (2002). Going with the flow or life in the fast lane: Contrasting mitochondrial responses to thermal change. J. Exp. Biol. 205, 2237–2249. 10.1242/jeb.205.15.2237 12110658

[B18] Guppy M. Withers P. (1999). Metabolic depression in animals: Physiological perspectives and biochemical generalizations. Biol. Rev. 74, 1–40. 10.1017/s0006323198005258 10396183

[B19] Hazel J. R. (1995). Thermal adaptation in biological membranes: Is homeoviscous adaptation the explanation? Annu. Rev. Physiol. 57, 19–42. 10.1146/annurev.ph.57.030195.000315 7778864

[B20] Holenweg A. K. Reyer H. U. (2000). Hibernation behavior of *Rana lessonae* and *R. esculenta* in their natural habitat. Oecologia 123, 41–47. 10.1007/s004420050987 28308742

[B21] Jin L. Yu J. Yang Z. Merilä J. Liao W. (2018). Modulation of gene expression in liver of hibernating Asiatic toads (*Bufo gargarizans*). Int. J. Mol. Sci. 19, 2363. 10.3390/ijms19082363 30103470 PMC6121651

[B22] Karanova M. (2016). Identification of phosphoethanolamine and phosphoserine in the brain of the pond fish *Perccottus glehni* (Eleotridae, Perciformes, Dyb. 1877). Neurosci. Behav. Physiol. 46, 803–807. 10.1007/s11055-016-0314-x 26336738

[B23] Le Sage E. H. LaBumbard B. C. Reinert L. K. Miller B. T. Richards‐Zawacki C. L. Woodhams D. C. (2021). Preparatory immunity: Seasonality of mucosal skin defences and *Batrachochytrium* infections in Southern leopard frogs. J. Anim. Ecol. 90, 542–554. 10.1111/1365-2656.13386 33179786

[B24] Lee J. Giordano S. Zhang J. (2012). Autophagy, mitochondria and oxidative stress: Cross-talk and redox signalling. Biochem. J. 441, 523–540. 10.1042/BJ20111451 22187934 PMC3258656

[B25] Levine B. Mizushima N. Virgin H. W. (2011). Autophagy in immunity and inflammation. Nature 469, 323–335. 10.1038/nature09782 21248839 PMC3131688

[B26] Lin H. Rowlands D. Jr (1973). Thermal regulation of the immune response in South American toads (*Bufo marinus*). Immunology 24, 129–133. 4631060 PMC1422882

[B27] Lin J. Huang Y. Bian M. Wan Q. Fang S. (2020). A unique energy-saving strategy during hibernation revealed by multi-omics analysis in the Chinese alligator. Iscience 23, 101202. 10.1016/j.isci.2020.101202 32534442 PMC7298530

[B28] Livak K. J. Schmittgen T. D. (2001). Analysis of relative gene expression data using real-time quantitative PCR and the 2^−ΔΔCT^ method. Methods 25, 402–408. 10.1006/meth.2001.1262 11846609

[B29] Lu X. Li B. Li Y. Ma X. Fellers G. M. (2008). Pre-hibernation energy reserves in a temperate anuran, *Rana chensinensis*, along a relatively fine elevational gradient. Herpetol. J. 18, 97–102.

[B30] Ma X. Lu X. Merilä J. (2009). Altitudinal decline of body size in a Tibetan frog. J. Zool. 279, 364–371. 10.1111/j.1469-7998.2009.00627.x

[B31] Maniero G. D. Carey C. (1997). Changes in selected aspects of immune function in the leopard frog, *Rana pipiens*, associated with exposure to cold. J. Comp. Physiol. B 167, 256–263. 10.1007/s003600050072 9203367

[B32] Mizushima N. (2007). Autophagy: Process and function. Gene Dev. 21, 2861–2873. 10.1101/gad.1599207 18006683

[B33] Mo N. Zhu D. Liu J. Feng T. Cui Z. (2022). Metabolic responses to air-exposure stress of the Chinese mitten crab (*Eriocheir sinensis*) revealed by a combined analysis of metabolome and transcriptome. Aquaculture 548, 737710. 10.1016/j.aquaculture.2021.737710

[B34] Modica-Napolitano J. S. Renshaw P. F. (2004). Ethanolamine and phosphoethanolamine inhibit mitochondrial function *in vitro*: Implications for mitochondrial dysfunction hypothesis in depression and bipolar disorder. Biol. Psychiat. 55, 273–277. 10.1016/S0006-3223(03)00784-4 14744468

[B35] Nelson C. J. Otis J. P. Carey H. V. (2010). Global analysis of circulating metabolites in hibernating ground squirrels. Comp. Biochem. Physiol. D. 5, 265–273. 10.1016/j.cbd.2010.07.002 20728417

[B36] Nelson C. J. Otis J. P. Martin S. L. Carey H. V. (2009). Analysis of the hibernation cycle using LC-MS-based metabolomics in ground squirrel liver. Physiol. Genomics 37, 43–51. 10.1152/physiolgenomics.90323.2008 19106184

[B37] Niu Y. Cao W. Storey K. B. He J. Wang J. Zhang T. (2020). Metabolic characteristics of overwintering by the high-altitude dwelling Xizang plateau frog, *Nanorana parkeri* . J. Comp. Physiol. B 190, 433–444. 10.1007/s00360-020-01275-4 32274534

[B38] Niu Y. Cao W. Wang J. He J. Storey K. B. Ding L. (2021a). Freeze tolerance and the underlying metabolite responses in the Xizang plateau frog, *Nanorana parkeri* . J. Comp. Physiol. B 191, 173–184. 10.1007/s00360-020-01314-0 33025179

[B39] Niu Y. Cao W. Zhao Y. Zhai H. Zhao Y. Tang X. (2018). The levels of oxidative stress and antioxidant capacity in hibernating *Nanorana parkeri* . Comp. Biochem. Physiol. A 219, 19–27. 10.1016/j.cbpa.2018.02.003 29454142

[B40] Niu Y. Chen Q. Storey K. B. Teng L. Li X. Xu T. (2022). Physiological ecology of winter hibernation by the high-altitude frog *Nanorana parkeri* . Physiol. Biochem. Zool. 95, 201–211. 10.1086/718764 35175907

[B41] Niu Y. Zhang X. Zhang H. Xu T. Men S. Storey K. B. (2021b). Antioxidant and non-specific immune defenses in partially freeze-tolerant Xizang plateau frogs, *Nanorana parkeri* . J. Therm. Biol. 102, 103132. 10.1016/j.jtherbio.2021.103132 34863473

[B42] Niu Y. Zhang X. Zhang H. Xu T. Zhu L. Storey K. B. (2021c). Metabolic responses of plasma to extreme environments in overwintering Tibetan frogs *Nanorana parkeri*: A metabolome integrated analysis. Front. Zool. 18, 41–13. 10.1186/s12983-021-00428-7 34454525 PMC8403389

[B43] Pei J. Xu Y. Zong S. Ren L. (2021). Transcriptomic and metabolomic data reveal the key metabolic pathways affecting *Streltzoviella insularis* (Staudinger)(Lepidoptera: Cossidae) larvae during overwintering. Front. Physiol. 12, 655059. 10.3389/fphys.2021.655059 34220530 PMC8250450

[B44] Ren X. Yu Z. Xu Y. Zhang Y. Mu C. Liu P. (2020). Integrated transcriptomic and metabolomic responses in the hepatopancreas of kuruma shrimp (*Marsupenaeus japonicus*) under cold stress. Ecotox. Environ. Safe. 206, 111360. 10.1016/j.ecoenv.2020.111360 32979723

[B45] Rumschlag S. L. Boone M. D. (2018). High juvenile mortality in amphibians during overwintering related to fungal pathogen exposure. Dis. Aquat. Organ. 131, 13–28. 10.3354/dao03277 30324911

[B46] Salem M. Rexroad C. E. Wang J. Thorgaard G. H. Yao J. (2010). Characterization of the rainbow trout transcriptome using Sanger and 454-pyrosequencing approaches. BMC Genomics 11, 1–10. 10.1186/1471-2164-11-564 20942956 PMC3091713

[B47] Sandmeier F. C. Horn K. R. Tracy C. R. (2016). Temperature-independent, seasonal fluctuations in immune function of the Mojave Desert Tortoise (*Gopherus agassizii*). Can. J. Zool. 94, 583–590. 10.1139/cjz-2016-0010

[B48] Santana F. E. Swaisgood R. R. Lemm J. M. Fisher R. N. Clark R. W. (2015). Chilled frogs are hot: Hibernation and reproduction of the endangered mountain yellow-legged frog *Rana muscosa* . Endanger. Species Res. 27, 43–51. 10.3354/esr00648

[B49] Scapin S. Baldini P. Luly P. (1990). Phospholipid and fatty acid composition of frog (*Rana esculenta*) liver—A circannual study. Lipids 25, 443–449. 10.1007/BF02538086

[B50] Šíma P. Sýkora J. Pospíšil M. (1981). “Comparison of the proliferative activity of lymphoid spleen cells and the antibody response in *Rana esculenta* kept in 22 and 4°C,” in Aspects of developmental and comparative immunology (Elsevier), 497–498. 10.1016/B978-0-08-025922-2.50082-6

[B51] Singh R. Sinha R. (1989). Seasonal changes in energy reserves in the common frog, *Rana tigrina* . Jpn. J. Physiol. 39, 969–973. 10.2170/jjphysiol.39.969 2632907

[B52] Storey J. M. Storey K. B. (1985). Adaptations of metabolism for freeze tolerance in the gray tree frog, *Hyla versicolor* . Can. J. Zool. 63, 49–54. 10.1139/z85-009

[B53] Storey K. B. (1990). Biochemistry of natural freeze tolerance in animals: Molecular adaptations and applications to cryopreservation. Biochem. Cell Biol. 68, 687–698. 10.1139/o90-100 2222994

[B54] Storey K. B. Storey J. M. Brooks S. Churchill T. A. Brooks R. J. (1988). Hatchling turtles survive freezing during winter hibernation. P. Natl. Acad. Sci. USA. 85, 8350–8354. 10.1073/pnas.85.21.8350 PMC282428 3186730

[B55] Storey K. B. Storey J. M. (1986). Freeze tolerance and intolerance as strategies of winter survival in terrestrially-hibernating amphibians. Comp. Biochem. Physiol. A 83, 613–617. 10.1016/0300-9629(86)90699-7 2870854

[B56] Storey K. B. Storey J. M. (1990). Metabolic rate depression and biochemical adaptation in anaerobiosis, hibernation and estivation. Q. Rev. Biol. 65, 145–174. 10.1086/416717 2201054

[B57] Storey K. B. Storey J. M. (2004). Metabolic rate depression in animals: Transcriptional and translational controls. Biol. Rev. 79, 207–233. 10.1017/S1464793103006195 15005178

[B58] Storey K. B. Storey J. M. (2017). Molecular physiology of freeze tolerance in vertebrates. Physiol. Rev. 97, 623–665. 10.1152/physrev.00016.2016 28179395

[B59] Sun H. Zuo X. Sun L. Yan P. Zhang F. Xue H. (2018). Insights into the seasonal adaptive mechanisms of Chinese alligators (*Alligator sinensis*) from transcriptomic analyses. Aust. J. Zool. 66, 93–102. 10.1071/ZO18005

[B60] Sun Y. Xiong Z. Xiang X. Liu S. Zhou W. Tu X. (2015). Whole-genome sequence of the Tibetan frog *Nanorana parkeri* and the comparative evolution of tetrapod genomes. P. Natl. Acad. Sci. USA. 112, E1257–E1262. 10.1073/pnas.1501764112 PMC4371989 25733869

[B61] Tattersall G. J. Ultsch G. R. (2008). Physiological ecology of aquatic overwintering in ranid frogs. Biol. Rev. 83, 119–140. 10.1111/j.1469-185X.2008.00035.x 18429765

[B62] Vistro W. A. Zhang Y. Bai X. Yang P. Huang Y. Qu W. (2019). *In vivo* autophagy up-regulation of small intestine enterocytes in Chinese soft-shelled turtles during hibernation. Biomolecules 9, 682. 10.3390/biom9110682 31683886 PMC6920937

[B63] Voituron Y. Hérold J.-P. Grenot C. (2000). Metabolic adaptations of overwintering European common lizards (*Lacerta vivipara*). Physiol. Biochem. Zool. 73, 264–270. 10.1086/316742 10893165

[B64] Wright R. K. Cooper E. L. (1981). Temperature effects on ectotherm immune responses. Dev. Comp. Immunol. 5, 117–122. 10.1016/0145-305X(81)90016-1

[B65] Wu Q. Sugimoto K. Moriyama K. Adachi Y. Nakayama A. Mori K. J. (2002). Cloning of hibernation-related genes of bullfrog (*Rana catesbeiana*) by cDNA subtraction. Comp. Biochem. Physiol. B 133, 85–94. 10.1016/S1096-4959(02)00117-3 12223215

[B66] Zhang J. Cai R. Liang J. Izaz A. Shu Y. Pan T. (2021). Molecular mechanism of Chinese alligator (*Alligator sinensis*) adapting to hibernation. J. Exp. Zool. B 336, 32–49. 10.1002/jez.b.23013 33231934

[B67] Zimmerman S. L. Frisbie J. Goldstein D. L. West J. Rivera K. Krane C. M. (2007). Excretion and conservation of glycerol, and expression of aquaporins and glyceroporins, during cold acclimation in Cope's gray tree frog *Hyla chrysoscelis* . Am. J. Physiol-Reg. I. 292, R544–R555. 10.1152/ajpregu.00434.2006 16973932

