# Supplementary material for: Integrated analysis of transcriptome and metabolome data reveals insights for molecular mechanisms in overwintering Tibetan frogs, Nanorana parkeri
Source: Front Physiol. 2023 Jan 9;13:1104476. doi: 10.3389/fphys.2022.1104476 (PMC9868574; doi:10.3389/fphys.2022.1104476)
Supplement: Supplementary file 2 [file DataSheet1.doc]

Table S1. Morphological parameters of *N. parkeri* in this study

|  | Summer | Winter |
| --- | --- | --- |
| Mean body mass (g) | 4.26 ± 0.04 | 4.41 ± 0.06 |
| Mean snout-vent length (cm) | 4.10 ± 0.05 | 4.05 ± 0.04 |

Note: the morphological data are expressed as the means ± SEM (n = 13).

Table S2. Primer sequences used in this study

| Genes | Primers Sequence (5’ to 3’) |
| --- | --- |
| ACO2(F) | CTGTTCCTGACACTGCTCGTTA |
| ACO2(R) | CAGTCCTTGTTTCTTTAGGTTGG |
| ASS1(F) | TCGCTCGTGTATCACCAAATCC |
| ASS1(R) | CATAATCTCCCTGCTTGTCCAT |
| HSPA9(F) | TAGTCGGTGGAATGACCAGAATG |
| HSPA9(R) | TACACCACCTTGAATAGCAGCAC |
| GDH(F) | TGGTCGCCTAACCTTCAAATA |
| GDH(R) | GCCAAGCCAGAGTGGACAATA |
| MDH(F) | TTTCAGATGGGAACTCCTATGG |
| MDH(R) | CCTCTTGAAGTTCTTTGGCTGT |
| PDHA1(F) | GCAGCCAGCACAGACTACTACA |
| PDHA1(R) | TAACTCCAGGGTCGCTCATACT |
| SDHA(F) | CTGCCAGGACTTGGAGTTTGT |
| SDHA(R) | TGTAGTGGACTGTGGGGAGGAC |
| SERP1(F) | CGAATGGCGAATGAGAAACAC |
| SERP1(R) | AACCAGGGACCTACCGATGAC |
| SERPIND1(F) | TATAGTGTCAATGGGTACAAAAGGG |
| SERPIND1(R) | TCTGCGGAAGAGTCTGTGAGTTA |
| SERPINF2(F) | TATAGCTGCTCGCATCTACGTC |
| SERPINF2(R) | CAACCTCTTCTGGGATACTCGTC |
| SIRT4(F) | GACTGTATGCCAGGACTGAACG |
| SIRT4(R) | GCTTATGGGATGAAAACTGTGAC |
| SIRT6(F) | TTCCAAAGTTAGAGGTCTACGGT |
| SIRT6(R) | GTTCCAAGTGTTATCGCCAAATC |
| TRPV4(F) | GAACTAAACAAGGCACCTCACC |
| TRPV4(R) | GATTTGGCTATCCTCTTTCTTCC |
| GAPDH(F) | GTTCTGGTGCCGACTATGTGG |
| GAPDH(R) | CAGGAGGCATTGCTGATAACTT |

Table S3. Summary of RNA-seq quality data of 6 samples in *N. parkeri*.

| Samples | Clean reads | Clean bases | Q20(%) | Q30 (%) | GC Content (%) | Map rate (%) |
| --- | --- | --- | --- | --- | --- | --- |
| S_liver_1 | 47,947,506 | 7,192,125,900 | 96.49 | 91.6 | 45.25 | 88.89 |
| S_liver_2 | 47,750,450 | 7,162,567,500 | 96.6 | 91.79 | 45.54 | 82.56 |
| S_liver_3 | 48,669,806 | 7,300,470,900 | 96.72 | 92.12 | 45.14 | 92.34 |
| W_liver_1 | 59,406,266 | 8,910,939,900 | 95.18 | 89.14 | 46.67 | 82.81 |
| W_liver_2 | 56,631,522 | 8,494,728,300 | 95.51 | 89.8 | 46.45 | 83.75 |
| W_liver_3 | 54,844,560 | 8,226,684,000 | 95.84 | 90.49 | 46.75 | 85.03 |

**Supplementary Figure Legends**

Fig. S1 Heat map diagram of correlations between the biological replicates. Pearson’s correlation coefficient was applied to evaluate reproducibility of biological replicates.

Fig. S2 The volcano plot shows the differentially expressed genes (DEGs) from transcriptomic analysis, the DEGs were screened through the FDR (False Discovery Rate) < 0.01 and |log_2_FoldChange| > 1 and highlighted with green (down regulated) and red (up regulated) colors.

Fig. S3 The top 20 enriched GO terms (biological process (A), cellular component (B), and molecular function (C)) for up-regulated genes in liver of winter-collected *N. parkeri* compared with summer-collected individuals (*P* < 0.05), the color shades represent different *P* values.

Fig. S4 The top 20 enriched GO terms (biological process (A), cellular component (B), and molecular function (C)) for down-regulated genes in liver of winter-collected *N. parkeri* compared with summer-collected individuals (*P* < 0.05), the color shades represent different *P* values.

Fig. S5 The top 20 enriched KEGG pathway for up- (A) and down-regulated (B) genes in liver of winter-collected *N. parkeri* compared with summer-collected individuals (*P* < 0.05), the color shades represent different *P* values.

Fig. S6 Heat map diagrams of DEGs involved in immune response (A) and phagosome (B) between two seasons. Red color indicates highly expressed genes, and blue indicates low expressed genes.

Fig. S7 Heat map diagrams of DEGs involved in damage repair mechanisms (antioxidant defense, endocytosis, lysosome, and autophagy). Red color indicates highly expressed genes, and blue indicates low expressed genes.

Fig. S8 Heat map diagrams of DEGs involved in the pathway of primary bile acid biosynthesis (A), glycolysis/gluconeogenesis (B), TCA cycle (C), oxidative phosphorylation (D), fatty acid metabolism (E), and amino acid metabolism. Red color indicates highly expressed genes, and blue indicates low expressed genes.

**Fig. S1**


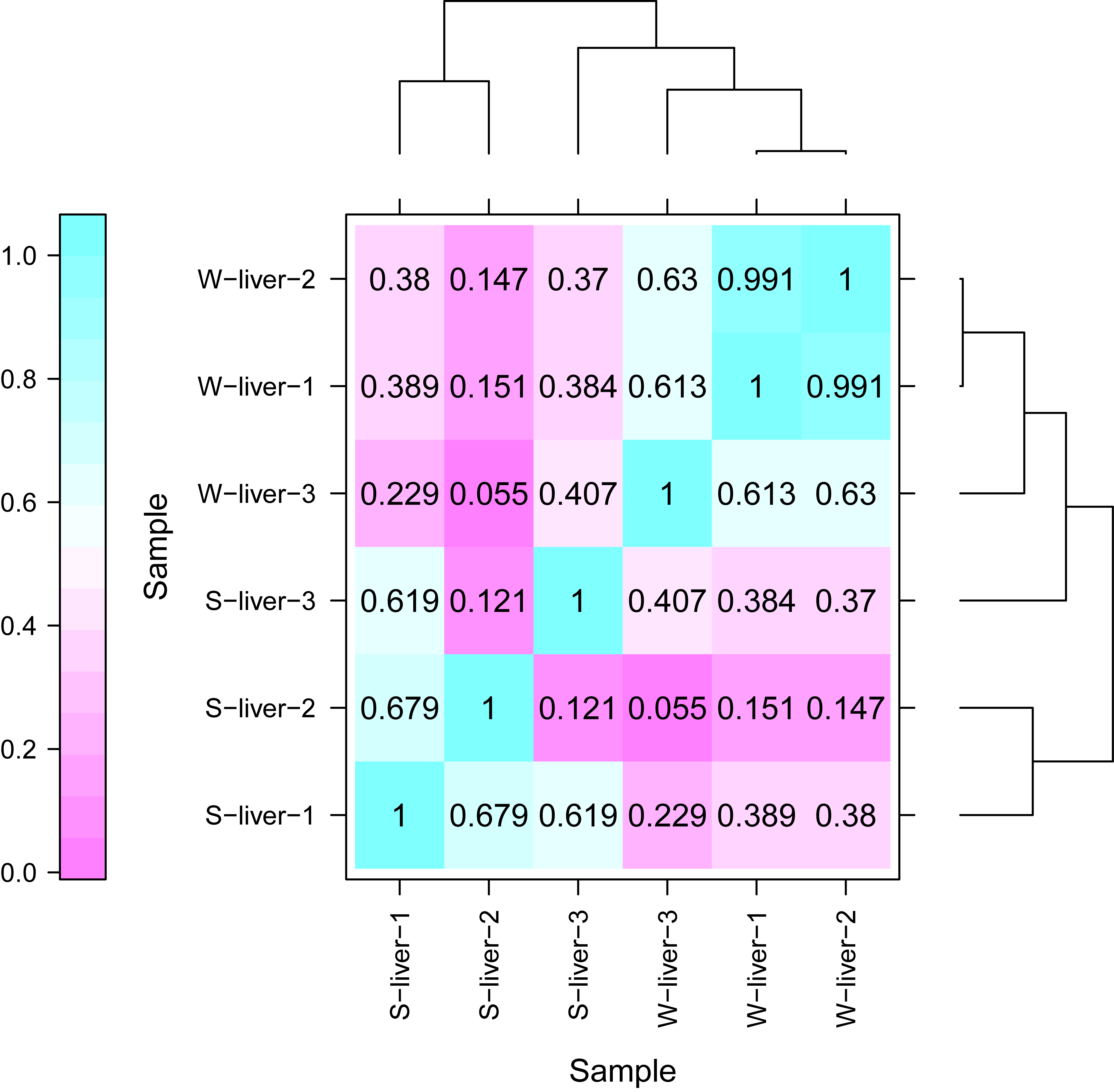


**Fig. S2**


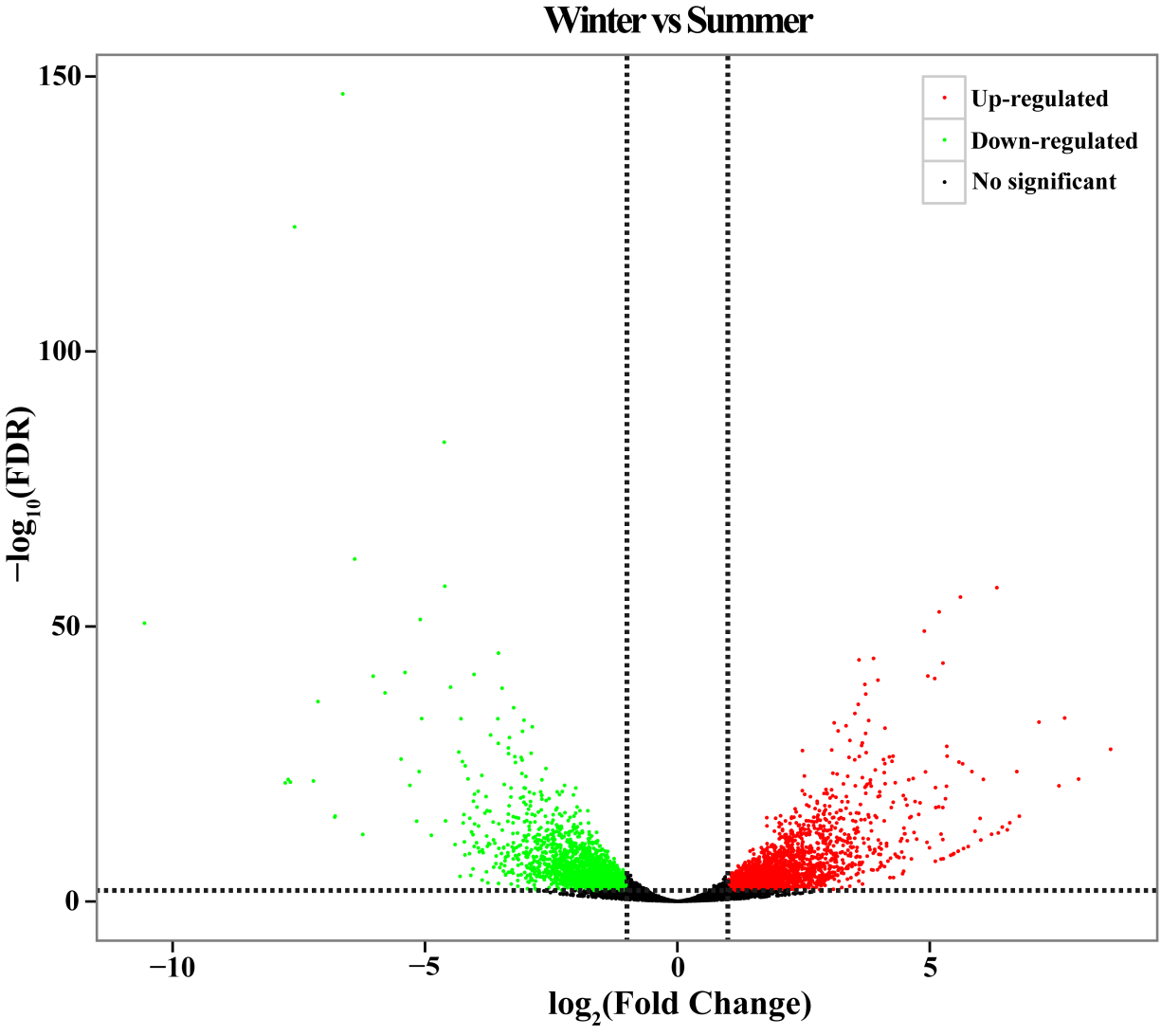


**Fig. S3**


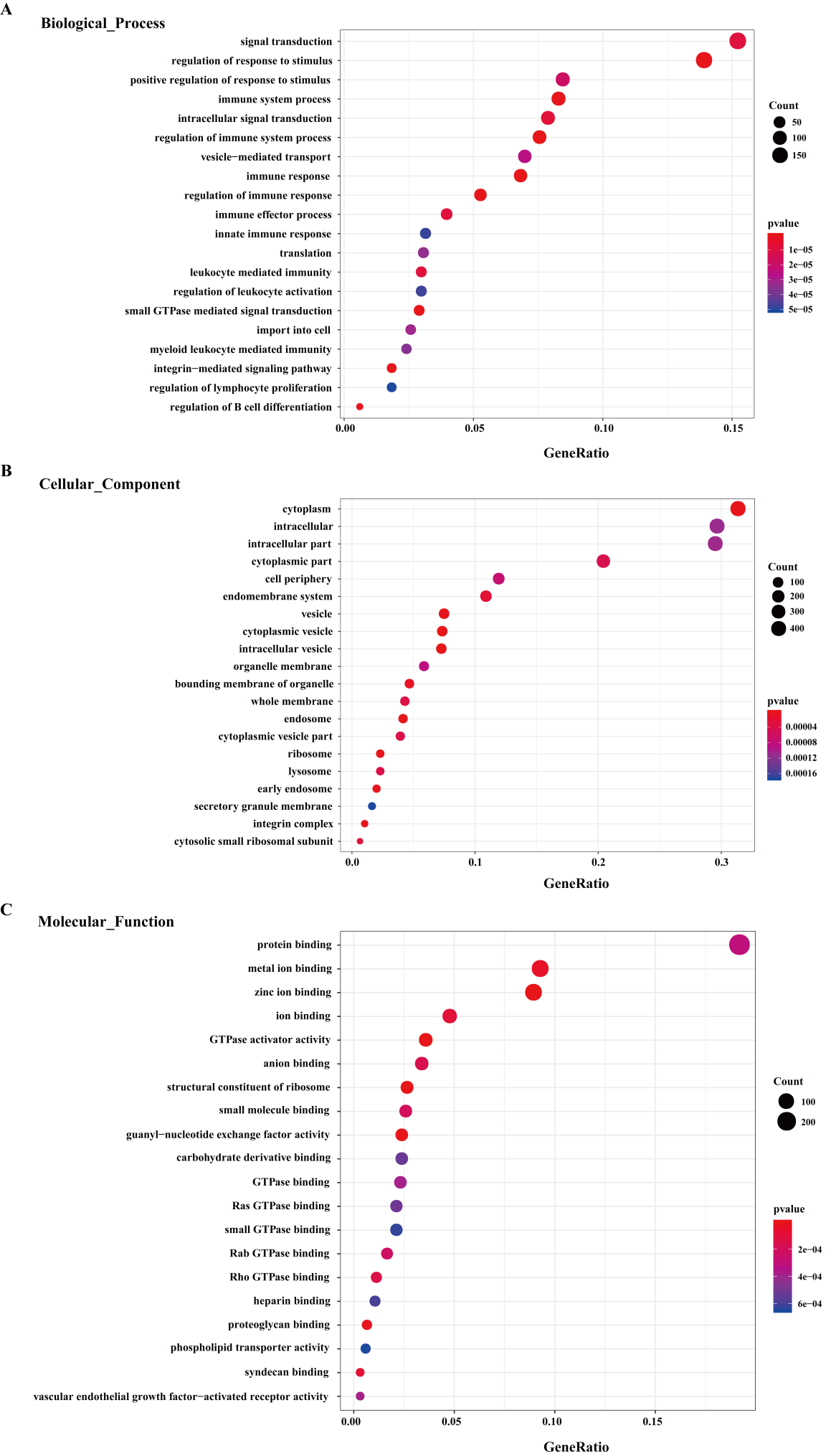


**Fig. S4**


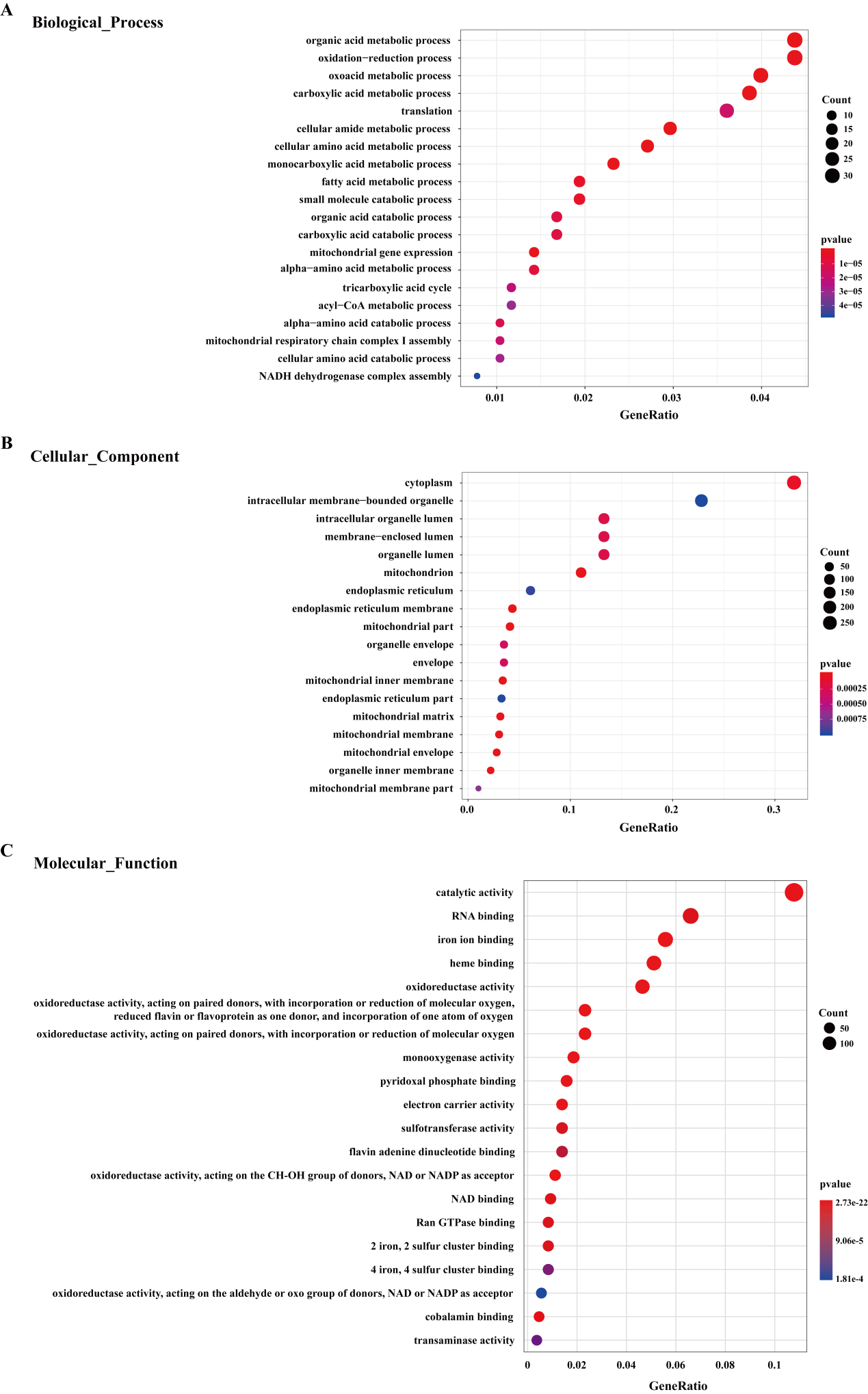


**Fig. S5**


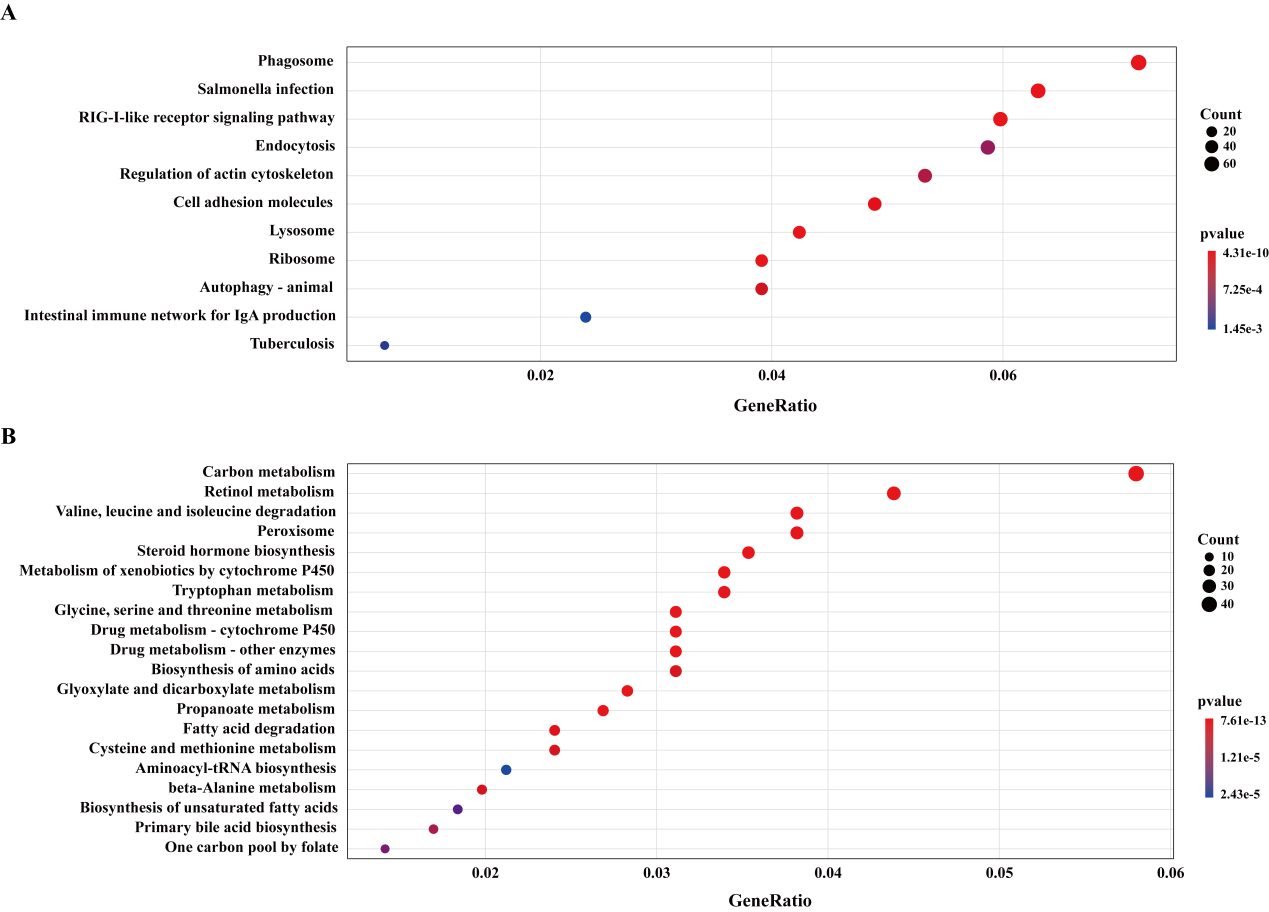


**Fig. S6**

**
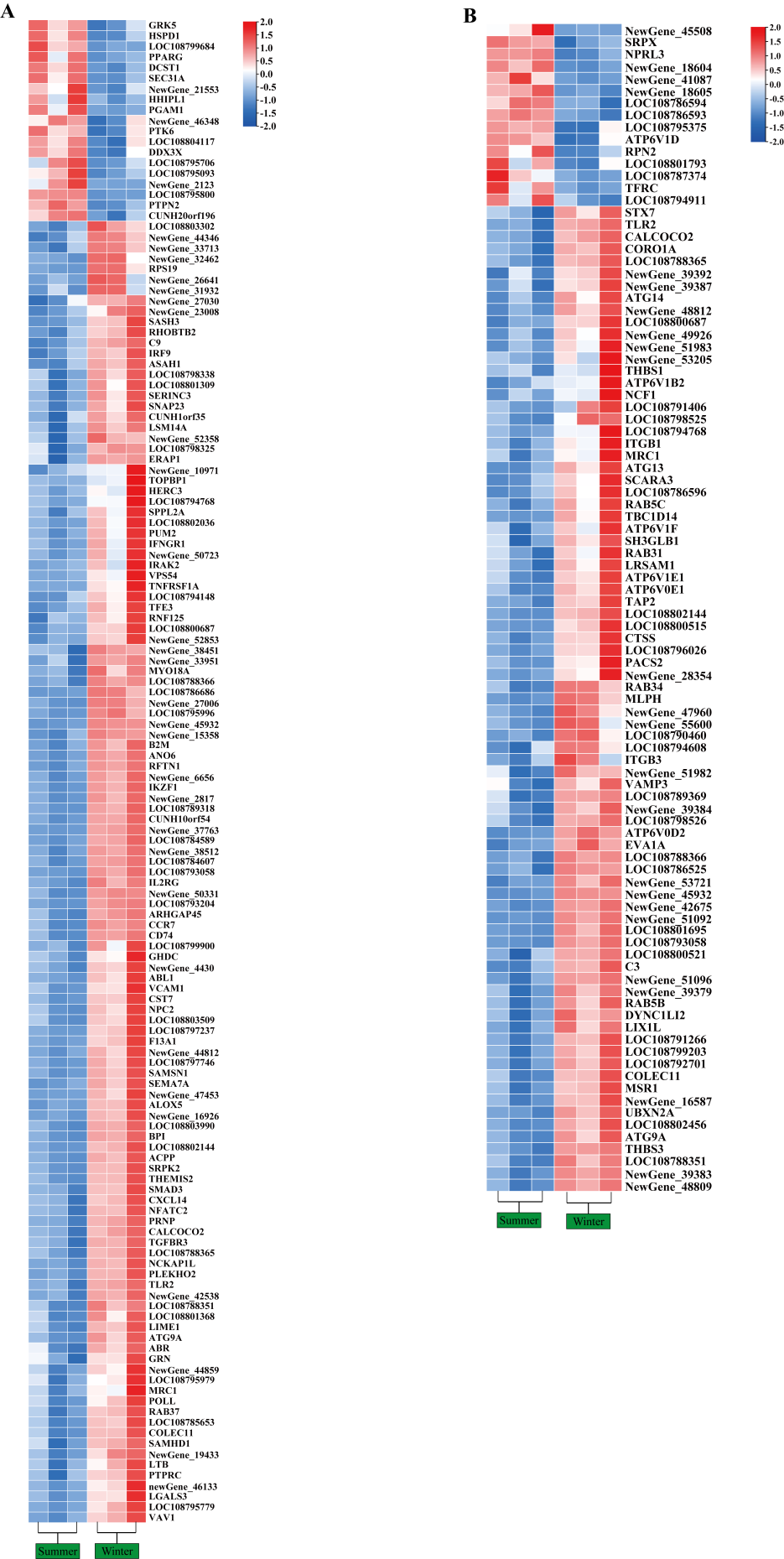
**

**Fig. S7**

**
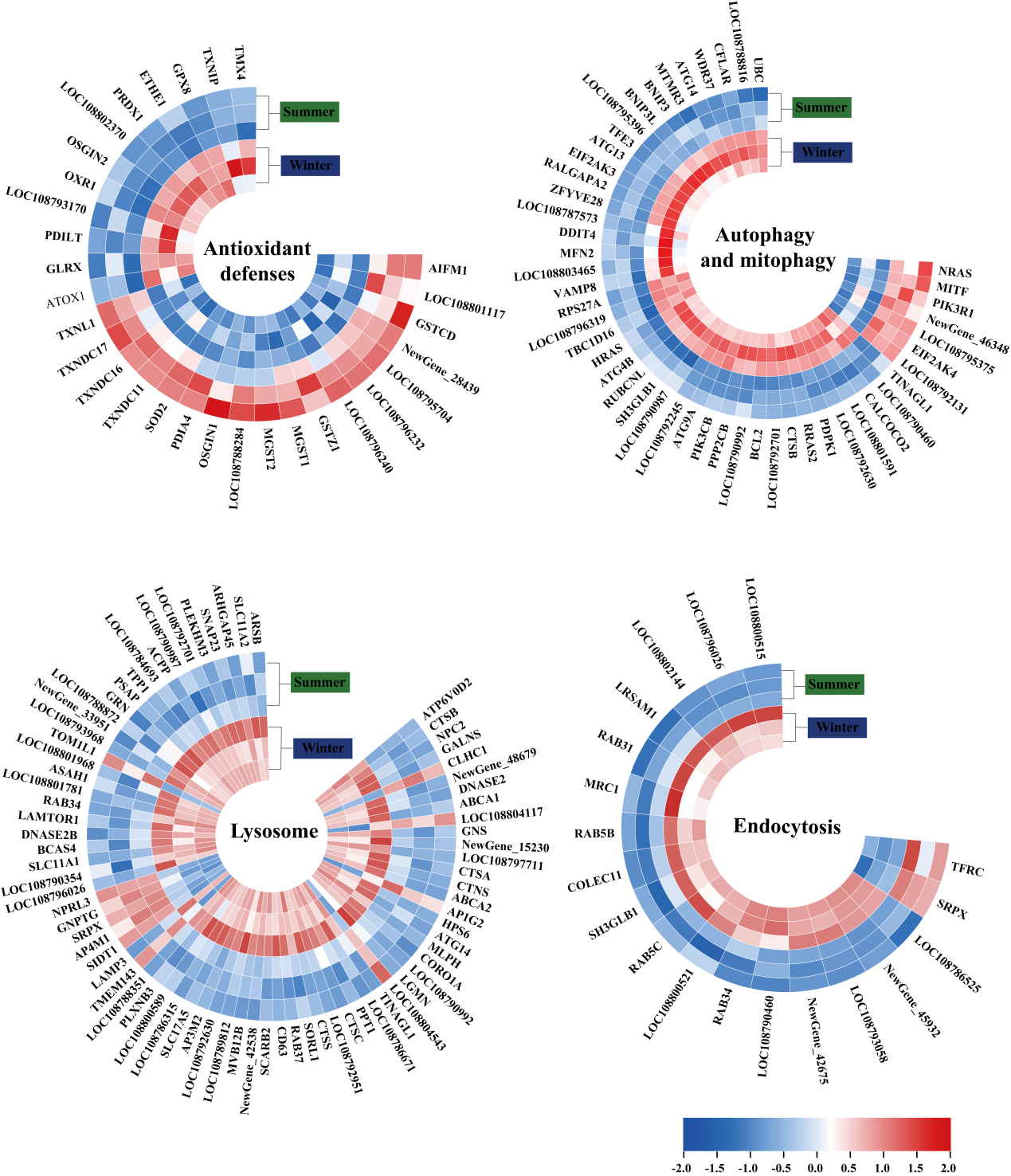
**

**Fig. S8**

**
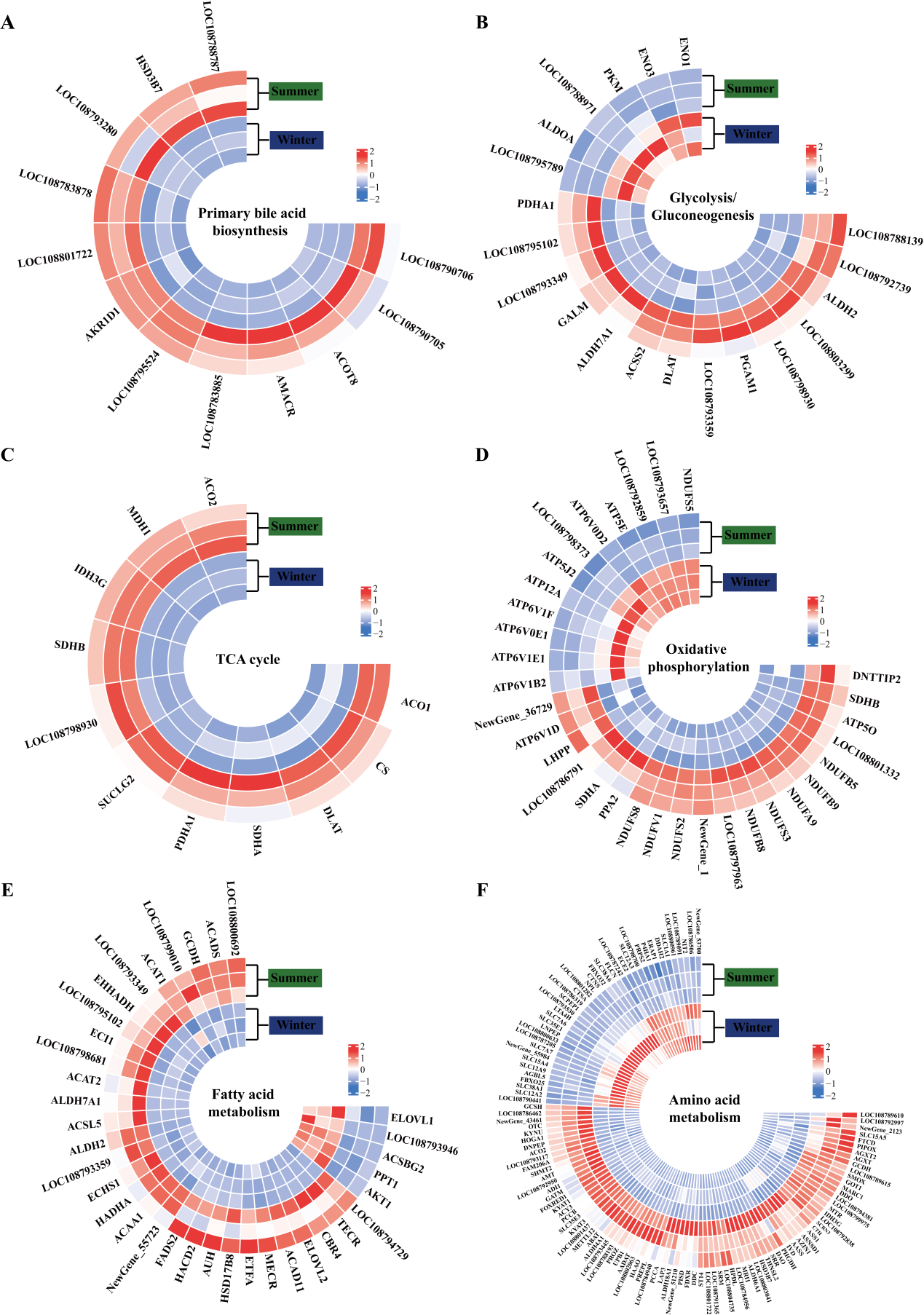
**
